# Binocular Integration in the Mouse Lateral Geniculate Nuclei

**DOI:** 10.1016/j.cub.2014.04.014

**Published:** 2014-06-02

**Authors:** Michael Howarth, Lauren Walmsley, Timothy M. Brown

**Affiliations:** 1 Faculty of Life Sciences, University of Manchester, Manchester M13 9PT, UK

## Abstract

A key task for the visual system is to combine spatially overlapping representations of the environment, viewed by either eye, into a coherent image. In cats and primates, this is accomplished in the cortex [1], with retinal outputs maintained as separate monocular maps en route through the lateral geniculate nucleus (LGN). While this arrangement is also believed to apply to rodents [2, 3], this has not been functionally confirmed. Accordingly, here we used multielectrode recordings to survey eye-specific visual responses across the mouse LGN. Surprisingly, while we find that regions of space visible to both eyes do indeed form part of a monocular representation of the contralateral visual field, we find no evidence for a corresponding ipsilateral representation. Instead, we find many cells that can be driven via either eye. These inputs combine to enhance the detection of weak stimuli, forming a binocular representation of frontal visual space. This extensive thalamic integration marks a fundamental distinction in mechanisms of binocular processing between mice and other mammals.

## Highlights

• The mouse LGN contains a binocular representation of frontal visual space
• Uncrossed retinal projections do not support exclusively monocular representations
• Nonlinear integration of binocular inputs enhances contrast detection

## Teaser

In cats and primates, binocular integration begins in the visual cortex. Howarth et al. show that in mice, eye-specific inputs are already extensively integrated by the level of the visual thalamus. This integration specifically enhances responses to weak stimuli within the frontal visual field.

## Results

### Binocular Response Types in the Mouse LGN

To assess eye-specific inputs to the visual thalamus we performed unilateral/bilateral multielectrode recordings across the lateral geniculate nucleus (LGN) of anesthetized mice (n = 39; 55 electrode placements). Based upon published anatomy [2, 3], we expected to find individual neurons responsive to either the contralateral or ipsilateral eye in the mouse dorsal LGN (dLGN; primary thalamocortical relay). We further aimed to determine whether a similar arrangement applies to LGN regions important for accessory visual function (intergeniculate leaflet and ventral part [IGL/vLGN]; [4, 5]).

**Figure 1:**
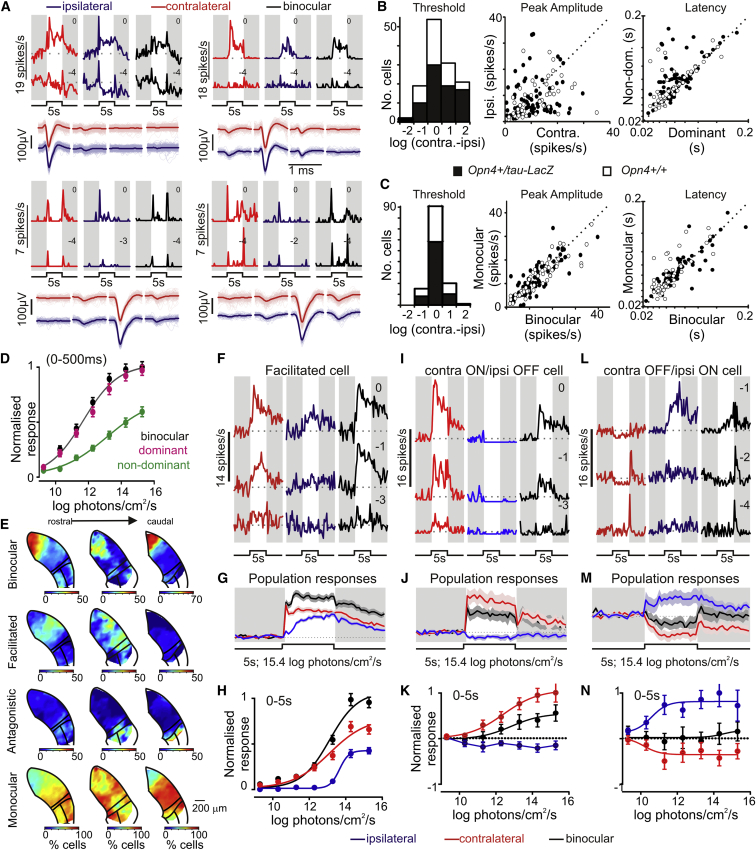
Binocular Visual Responses in Mouse LGN Neurons (A) Threshold and peak responses (means of ten trials) of four binocular LGN neurons to 5 s light steps applied to one or both eyes (410 nm light-emitting diode; numbers above traces indicate log intensity relative to the maximum: 15.4 log photons/cm^2^/s). Dotted lines in each trace indicate the mean prestimulus firing (0–5 s before step). Bottom traces represent “virtual” tetrode spike waveforms for each unit during stimuli restricted to either the ipsilateral or contralateral eye. Thick line represents the mean spike shape. (B) Relative log threshold intensity (left), peak amplitude (middle), and latency (right) for contralateral versus ipsilateral evoked responses of 127 binocular neurons (77 *Opn4*^+/tau-lacZ^ and 50 *Opn4*^+/+^ littermates). (C) Analysis as above for binocular versus dominant-eye-driven responses. (D) Mean ± SEM normalized responses (0–500 ms after light step) as a function of stimulus irradiance. Sigmoid fit coefficients for binocular and dominant-eye-only stimuli were statistically indistinguishable (F test, p = 0.07). (E) Relative proportion of visually responsive cells showing various binocular or monocular response profiles as a function of anatomical localization within the LGN. (F–N) response properties of facilitated (F–H) and antagonistic (I–K, contralateral ON/ipsilateral OFF; L–N, contralateral OFF/ipsilateral ON) cells. (F, I, and L) Example single-cell responses, with conventions as in (A). (G, J, and M) Mean ± SEM normalized population responses (n = 107, 18, and 22, respectively) to bright light steps targeting one or both eyes. (H, K, and N) Mean ± SEM normalized irradiance response functions (0–5 s after light step). See also Figure S1 for further details of unit isolation, spike waveforms, additional data, and anatomical distributions.

To help distinguish between dLGN and IGL/vLGN recordings, we performed a subset of these (n = 27; 36 electrode placements) in *Opn4*^+/tau-lacZ^ mice [6, 7] in which the IGL is readily detected by β-galactosidase staining (Figure S1A and S1B available online). Additional recordings from wild-type littermates (n = 19 placements in 12 mice) produced essentially identical results (Figures 1B, 1C, S1C, and S1D). Accordingly, we have combined these data sets for subsequent analysis.

We first recorded LGN responses to full-field stimuli (410 nm light-emitting diode; 5 s) applied at varying intensity to one or both eyes. As expected, most visually responsive LGN cells (n = 548/822) were driven exclusively by the contralateral retina (Figurea S1C, S1D, and S1N–S1P). Surprisingly, however, none of the remaining cells exhibited purely ipsilateral visual responses. Instead these displayed varying types of binocular interaction, of which the most common form (n = 127/274) was a brisk increase in firing elicited by stimulation of either eye (Figure 1A). These “binocular” cells were found at highest density around zones of anatomically defined ipsilateral retinal projections (Figures 1E and S1P; mediodorsal portion of the dLGN and the IGL/vLGN).

The relative magnitude of responses to stimulation of either eye varied for binocular cells (Figures 1A and 1B), but latencies were typically very similar, suggesting a direct convergence of ipsilateral/contralateral retinal ganglion cells (RGCs) onto the same LGN neuron. The monocular components were also markedly subadditive (Figures 1C and 1D), such that there was no clear response enhancement on binocular stimulation.

These properties rule out two trivial interpretations of our data. First, subadditive binocular responses exclude inadequate unit isolation (in which case the responses should be approximately additive; see also Figures 1A and S1G). Second, we didn’t observe the large differences in response threshold (Figures 1B and 1D) expected if one of the components resulted from insufficient optical isolation of our stimuli. To further rule out a contribution of off-target effects, we performed additional LGN recordings after retinal inactivation by intravitreal tetrodotoxin injection (Figures S1E and S1F). In no case did we observe any response appearing to originate from the injected eye.

We also observed many LGN neurons (n = 107) with contralateral-driven responses whose activity was modulated by bright stimuli presented to the ipsilateral eye (Figures 1F–1H). In these “facilitated” cells (most commonly found ventrolaterally in the dLGN; Figure 1E), monocular responses partially summed, leading to enhanced steady state firing upon binocular stimulation. Here, ipsilateral responses exhibited unusually sluggish kinetics (Figure S1L), suggesting that they were not directly driven by RGC inputs.

We also observed cells with “antagonistic” binocular responses (Figures 1I–1N), i.e., contralateral ON/ipsilateral OFF (n = 18) or the converse (n = 22). In a few cases, the inhibitory (OFF) component was not visible under monocular stimulation, but only as a dramatically reduced ON response under binocular stimulation (Figure S1M; n = 4). These antagonistic cells were only found at high density within the IGL/vLGN region (Figure 1E), forming a very small proportion of the dLGN neurons sampled (Figures S1O and S1P; ∼3% of the total). We have thus focused the remainder of our analysis on binocular and facilitated cells.

### Contrast and Irradiance Coding Properties

Since we hypothesized that the activity of binocular and facilitated cells might differentially depend on the absolute versus relative brightness of our stimuli (irradiance versus contrast), we next employed a protocol that allowed us to dissociate components of the response dependent on these features (see the Supplemental Experimental Procedures).

**Figure 2:**
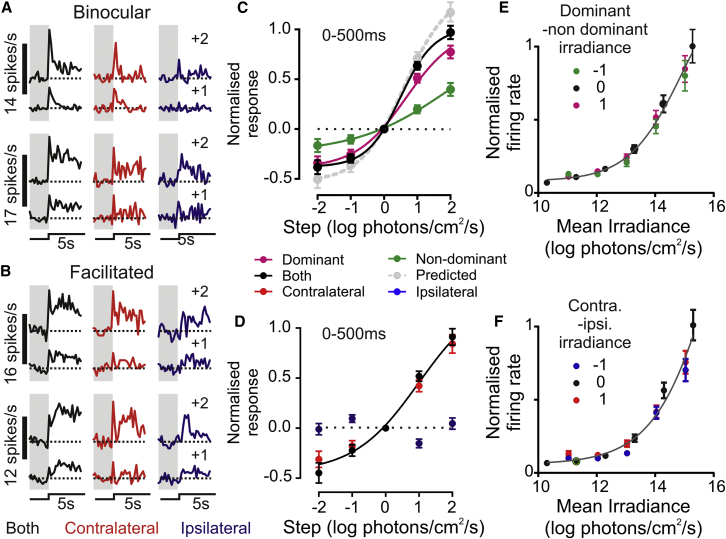
Binocular Input Enhances Visual Coding in LGN Cells (A and B) Example responses of two binocular (A) and two facilitated (B) cells to 1–2 log unit steps up in intensity targeting one or both eyes (means of 10–60 trials). Dotted lines indicate the mean basal firing rate 0–3 s before step. (C and D) Mean ± SEM normalized contrast response relationships of binocular (C; n = 97) and facilitated (D; n = 84) cells to stimuli applied to one or both eyes (light-adapted conditions: 11.4–14.4 log photons/cm^2^/s). The dotted gray line indicates the linear prediction for the binocular response (contralateral and ipsilateral). To facilitate comparison of cells with different response polarities on the same graph, we sign inverted ON inhibitions and OFF excitations. Binocular cell responses were best fit by separate four-parameter sigmoidal functions (F test, p < 0.001). Facilitated cell responses to contralateral and binocular stimuli were not significantly different (F test, p = 0.29). (E and F) Relationship between normalized steady state firing (mean ± SEM) of irradiance coding binocular (E; n = 57) and facilitated (F; n = 84) cells and mean binocular irradiance, plotted according to the difference in interocular irradiance. All data are well described by single four-parameter sigmoid functions regardless of the relative irradiance (F test, p = 0.34 and 0.27 in E and F, respectively). See also Figure S2 for additional analysis and responses of monocular irradiance coding cells.

We were particularly interested to understand the properties of binocular cells. We reasoned that the lack of binocular summation after light steps applied from darkness may have resulted from the unusually high contrast of these stimuli. To this end, we calculated contrast response relationships for stimuli applied to one or both eyes under light adapted conditions. We found the slope of these curves was indeed significantly steeper under binocular versus dominant-eye-only stimulation (Figures 2A and 2C; F test, p < 0.05). Extrapolating from these curves, the step in light intensity evoking a 50% maximal response was reduced from ∼11-fold to <5-fold under binocular versus dominant-eye-only stimulation. Thus, at least one function of LGN binocular integration is to enhance detection of weak stimuli.

By distinction, the acute response of facilitated cells was solely determined by the contralateral retina (Figures 2B and 2D); contrast responses were identical under binocular and contralateral-only stimulation (Figure 2D), and ipsilateral responses were essentially absent. An influence of the ipsilateral eye did become visible over later components of the response, however, (Figure 2B) even after modest steps in light intensity.

**Figure 3:**
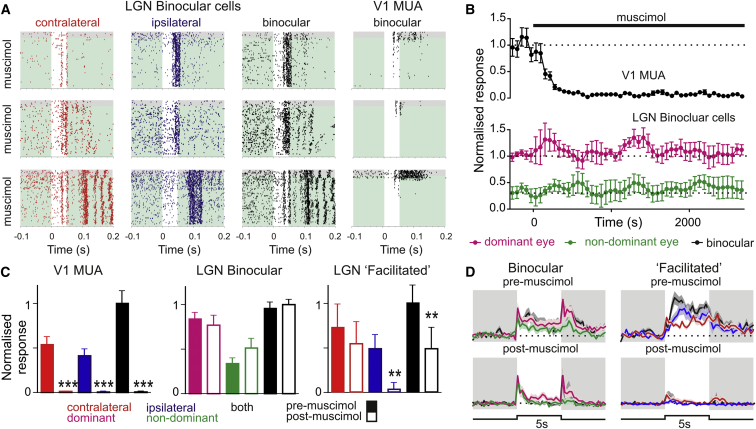
Cortical Feedback Is Not Required for LGN Binocular Cell Responses (A) Peristimulus rasters for three LGN binocular cell responses to flashes (50 ms; 15.4 log photons/cm^2^/s) targeting one or both eyes. Stimuli run from 5 min before to 45 min after cortical inactivation by topical application of 1 mM muscimol. The far-right panels are representative of simultaneously recorded V1 multiunit responses. Note that LGN binocular responses persist long after cortical responses disappear, even when there is a large difference in latency between eye-specific responses (bottom). (B) Time course of muscimol effects (mean ± SEM normalized response) on V1 multiunit response (n = 110 channels) and LGN binocular cell responses (n = 14). (C) Mean ± SEM V1 multiunit responses (left; n = 110 channels), LGN binocular cell responses (middle; n = 14), and facilitated cell responses (right; n = 9) across 5 min epochs before muscimol application (filled bars) and 40–60 min after (open bars). Data were analyzed by paired t test (^∗∗^p < 0.01, ^∗∗∗^p < 0.001). (D) Mean ± SEM normalized response of binocular (left) and facilitated (right) cells to 5 s light steps (15.4 log photons/cm^2^/s) before and after cortical inactivation. The dotted line indicates the mean prestimulus (0–5 s) firing. Note that although transient contrast responses persist, sustained response components are attenuated after muscimol application. See also Figure S3 for spike waveforms and example facilitated cell responses.

One interpretation of these data is that the basal activity of facilitated cells provides information about binocular irradiance. Accordingly, we next calculated their steady-state firing as a function of absolute irradiance. Applying an information-theory-based test (see the Supplemental Experimental Procedures), we determined that the basal firing activity of each of these facilitated cells did indeed encode significant (p < 0.05) information about binocular irradiance. Accordingly, when plotted as a function of the average irradiance across the two eyes, steady-state firing was well described by a single sigmoid curve regardless of the interocular difference in brightness (F test, p = 0.27; Figure 3F). The same was not true if instead these data were instead expressed solely in terms of contralateral irradiance (Figure S2B).

This irradiance coding property also extended to many binocular cells (n = 57/91). Activity of this subset of cells was also well described by a single curve when expressed in terms of mean binocular irradiance (Figure 2E; F test, p = 0.34), but not when quantified in terms of irradiance at the dominant eye (Figure S2A). These properties were not a general feature of irradiance coding LGN neurons, however, since we identified many monocular LGN cells (n = 175/363) that solely encoded contralateral irradiance (Figure S2C).

### Role of Cortical Feedback

In addition to direct retinal input, a major source of excitatory input to the dLGN comes via feedback from the visual cortex [8, 9]. To establish whether corticothalamic feedback contributed to binocular responses in the LGN, in some experiments (n = 7) we assessed LGN visually evoked activity (50 ms flashes; 15.4 log photons/cm^2^/s) after cortical inactivation by topical application of muscimol (GABA_A_ agonist). As previously reported [10], muscimol produced a profound and persistent inhibition of cortical activity, essentially abolishing firing by 40–60 min (Figures 3A–3C; 99.1% ± 0.7% block). Responses of simultaneously recorded LGN binocular cells remained unchanged, however (Figures 3A–3C; n = 14). This was even true of one cell (Figure 3A) in which ipsilateral responses were significantly slower than those driven by contralateral stimulation.

By contrast, all ipsilateral components of facilitated cell responses disappeared after cortical inactivation (n = 9; Figures 3C and S3B). A further effect was revealed when we then switched to longer (5 s) light steps (45–90 min postmuscimol). While contralateral stimuli still evoked transient increases in firing, all sustained components of the responses of facilitated cells were completely abolished (Figure 3D). We also observed a reduction in sustained responses of binocular cells (Figure 3D), suggesting that the binocular irradiance coding properties described above may rely on cortical feedback.

### Spatial Response Properties

Our data above suggest that binocular integration enhances LGN visual coding. One condition of our interpretation is that both monocular inputs should originate from equivalent regions of visual space. To confirm this and to rule out the possibility that our use of full-field stimuli biased our recordings toward a subset of LGN neurons (see the Supplemental Experimental Procedures), we also examined spatial response properties in a subset of bilateral recordings (n = 9).

**Figure 4:**
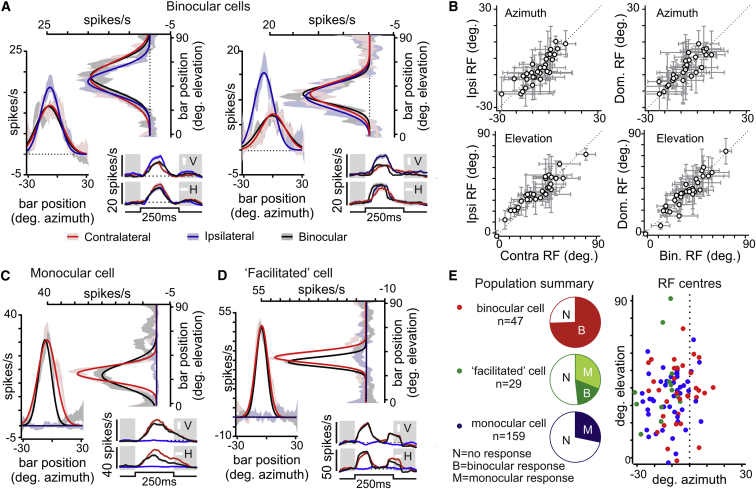
LGN subpopulations encode binocular or monocular representations of frontal visual space. (A) RF maps for two binocular neurons obtained using flashing horizontal and vertical white bars under either monocular or binocular viewing conditions. Solid lines indicate Gaussian fit, shaded areas indicate mean ± SEM firing response (eight trials), and angles are expressed relative to the skull’s midpoint. The inset shows mean ± SEM response to optimal horizontal or vertical bars (10 ms bins, 100 ms boxcar smoothing). The dotted line indicates the mean baseline firing rate (0–125 ms before bar appearance). (B) Ipsilateral versus contralateral (left) and dominant-eye versus binocular (right) RF correspondence for 30 binocular cells. Error bars represent the estimated RF radius. (C and D) RF maps for monocular (C) and facilitated (D) cells, with conventions as in (A). (E) Summary of population data showing proportion of cells exhibiting binocular/monocular RF properties (left) and RF center positions (right) for all responding neurons (n = 35, 14, and 45 for binocular, facilitated, and monocular cells, respectively). See also Figure S4 for additional examples, analysis, and retinotopic organization.

To localize the receptive field (RF) centers for binocular cells, we applied horizontal and vertical bar stimuli via a display centered within frontal visual space. Since this display encompassed >70% of the binocular zone, we predicted that it should be possible to determine RF locations for a similar proportion of binocular cells. Accordingly, with both eyes viewing, this was indeed possible for 35/47 binocular cells (Figures 4A, 4B, and 4E). RFs varied in diameter and spatial location (Figures 4B and S4B) and the majority were of the ON variety (n = 24; Figures 4A and S4F), although we also observed OFF (n = 6; Figure S4D) and ON-OFF (n = 5) responses.

We next determined eye-specific contributions to the binocular RFs. Of the 35 cells with RFs within the region surveyed, 30 also responded when stimulation was restricted to either eye alone (Figures 4A, 4B, S4D, and S4E). The signs (ON or OFF) of these monocular RFs were invariably consistent, while the relative amplitudes varied from cell to cell. We also observed modest differences in RF position and/or diameter (Figure 4B; mean ± SEM: azimuth, 3.8° ± 0.5°; elevation, 5.7° ± 0.5°; width, 5.0° ± 1.5°). However, our estimates of such differences were markedly less than the size of the dominant eye RF (median ± SD: 17.6° ± 8.9°), such that eye-specific RFs occupied overlapping regions of visual space (Figure 4B).

We also observed one cell that responded only upon binocular stimulation, and there were four in which we could only map RFs for one eye (two ipsilateral, two contralateral). Each of these cells exhibited substantially larger responses after binocular stimulation than under monocular viewing (78% ± 22%; paired t test, p < 0.05). For the other cells, dominant-eye and binocular responses were of similar magnitude (17.3 ± 2.3 versus 15.2 ± 2.2 spikes/s, respectively; paired t test, p > 0.05). Similarly, there was no significant difference in RF size between binocular and monocular viewing conditions (mean ± SEM: 1.0° ± 1.5°; p > 0.05).

A smaller proportion of facilitated cells (14/29) had measureable RFs within the area of our visual display (Figures 4E and S4E), and only five cells showed evidence of a binocular response to flashing bars (weak ipsilateral responses or response to binocular only). The remaining cells exhibited purely contralateral visual responses that were similar under binocular and monocular viewing (Figure 4D). To probe for more sluggish responses, we also employed 0.2 Hz reversing gratings of various spatial frequencies. Since these did not reveal any clear binocular response (data not shown), we conclude that the binocular irradiance coding properties described above may operate on a more global scale than the contrast responses of facilitated cells.

Given the substantial binocular integration that we describe here, one might expect that monocular LGN cells would receive input from regions of space visible to only the contralateral eye. In fact, many monocular cells did respond to stimuli within the binocular zone (45/159; Figures 4C and 4E). None of these, however, responded to stimuli restricted to the ipsilateral retina, nor did we find any difference in response amplitude upon contralateral versus binocular viewing (18.0 ± 1.8 versus 16.8 ± 1.6 spikes/s). Thus, while there is no purely ipsilateral representation of binocular visual space, a contralateral-only representation is present within the mouse LGN.

Consistent with previous reports that RF size in the mouse LGN increases with visual eccentricity [11], RFs of all cell classes categorized here tended toward the larger end (Figure S4B) of those generally reported for LGN neurons (11°–17°; [11, 12]). Our analysis indicates that eye movements are unlikely to have been a significantly factor in our experiments (Figure S4C), nor did this finding appear to be an artifact of our RF mapping approach. Indeed, in some experiments (n = 5), we also employed sparse noise stimuli (similar to those used previously in the mouse [12]), and we found a good correlation to estimates of RF position and diameter obtained with bars (Figures S4D–S4H; see also the Supplemental Experimental Procedures).

Finally, we also confirmed that cells responding to our spatial stimuli exhibited the expected retinotopic location within the dLGN. This was the case for both binocular and monocular cells (Figures S4I–S4K), with more dorsomedially located cells preferring spatial stimuli closer to the midline and at greater elevations, as described previously [12, 13].

## Discussion

Unexpectedly, we find no evidence for functional segregation of ipsilateral visual signals in the mouse LGN. Instead, we find many cells exhibiting some form of binocular response. Most commonly, these manifest as rapid excitatory responses to contrast in a specific region of space that can be evoked via either eye independently. Integration of these binocular inputs is markedly subadditive for strong stimuli, but acts to increase contrast sensitivity, enhancing responses to low-contrast elements in the visual scene.

Given the generally near-identical latency of their monocular inputs, the most parsimonious explanation for our data is that many binocular cells receive monosynaptic input from either retina. This arrangement would be in stark contrast with the cat or primate LGN, in which binocular interactions (when present) are believed to arise polysynaptically [14–17]. An earlier study did find evidence for direct binocular integration in the rat LGN [18], however, suggesting this may be a common feature of rodent visual systems.

Direct binocular integration seems surprising, given the anatomical separation of LGN retinal inputs. It is of note, however, that the ipsilateral terminal field is relatively thin across most of the dLGN (∼100 μm; [2]) by comparison with the dendritic field of mouse thalamocortical projection neurons (∼150 μm; [19]). Accordingly, it would seem that only cells at the very center of the dLGN ipsilateral zone could be isolated from contralateral inputs. Indeed, anatomical studies have identified LGN relay cells whose dendritic fields span eye-specific domains [19].

Nonetheless, one would still expect to find some purely ipsilateral neurons. Although rare (1%–2%), we did find a few dLGN cells that exhibited excitatory responses to stimulation of the ipsilateral retina and inhibitory contralateral responses. We suspect direct RGC input to these cells is purely ipsilateral, with the contralateral inhibition provided via interneurons that span eye-specific domains [20]. We also found LGN binocular cells in which the latency difference between ipsilateral and contralateral responses was sufficiently large to allow for indirect integration. Based on our cortical inactivation experiments, it is unlikely that this could arise through top-down influences. Such integration could occur subcortically, however (e.g., [21]).

There have been few functional investigations of binocular integration in the mouse LGN. Binocular input to lateral margins of the dLGN is found only in immature rodents [22, 23], but these studies did not investigate the medial regions where we find binocular cells. Our findings are apparently at odds, however, with a previous report of exclusively ipsilateral mouse LGN neurons [13]. We suspect that this discrepancy relates to the higher contrast of our stimuli relative to this previous work (70% contrast gratings), which we predict would generally evoke weak responses when presented to the nondominant eye alone. Consistent with this view, another recent study using higher-contrast gratings has reported binocular responses in the mouse LGN [24].

Mouse cortical neurons exhibit varying preference toward either eye, hitherto assumed to derive from differential pooling of eye-specific monocular inputs [25–28]. Although our data indicate that much of this diversity is already present at the level of individual LGN neurons, it is also clear that further integration must occur in the cortex, most notably because dLGN RFs (present study; [10–13]) typically lack the strongly oriented structure of V1 simple cells. We find a purely contralateral representation of frontal visual space in the mouse dLGN, and it seems likely that this is combined with the equivalent binocular representation to generate the distribution of ocular dominance observed in cortical neurons.

In addition to conventional binocular integration, we also observed a cortically imposed binocular irradiance signal across many LGN cells. This signal is independent of the magnitude of eye-specific contrast responses and extends to facilitated cells located outside of what is traditionally considered the dLGN binocular zone. Although we have been unable to determine the spatial extent of this influence within individual neurons, given the anatomical distribution of cells exhibiting this property, we suspect that it operates across a more global spatial scale than the classical RF. Such global irradiance signals might provide important contextual information used at higher stages of the visual pathway, for example to infer surface brightness and/or lightness [29].

We also investigated whether binocular processing differed between LGN subregions serving cortical versus subcortical pathways. In this regard, we found a greatly increased prevalence of antagonistic binocular responses in the IGL/vLGN region, presumably driven by inhibitory connections within and/or between the nuclei of opposing hemispheres [4, 5]. Insofar as these cells provide information about interocular differences in irradiance, we speculate they may be important for visuomotor control, a key proposed function of the IGL/vLGN.

Together, our data reveal fundamental differences in the processing of eye-specific signals within the mouse LGN relative to other mammals. These findings have important implications for our understanding of ecological and evolutionary aspects of visual system organization and for the use of the mouse as a model to understand human vision. In particular, the widespread integration of eye-specific signals in the mouse LGN should be taken into account when investigating binocular responses further along the visual pathway.

## Supplemental Information

Document S1. Figures S1–S4 and Supplemental Experimental Procedures

Document S2. Article plus Supplemental Information
