# Supplementary material for: Binocular Integration in the Mouse Lateral Geniculate Nuclei
Source: Curr Biol. 2014 Jun 2;24(11):1241–7. doi: 10.1016/j.cub.2014.04.014 (PMC4046226; doi:10.1016/j.cub.2014.04.014)
Supplement: Document S1. Figures S1–S4 and Supplemental Experimental Procedures [file mmc1.pdf]

Current Biology, Volume *24*

Supplemental Information

Binocular Integration in the Mouse Lateral Geniculate Nuclei

Michael Howarth, Lauren Walmsley, and Timothy M. Brown

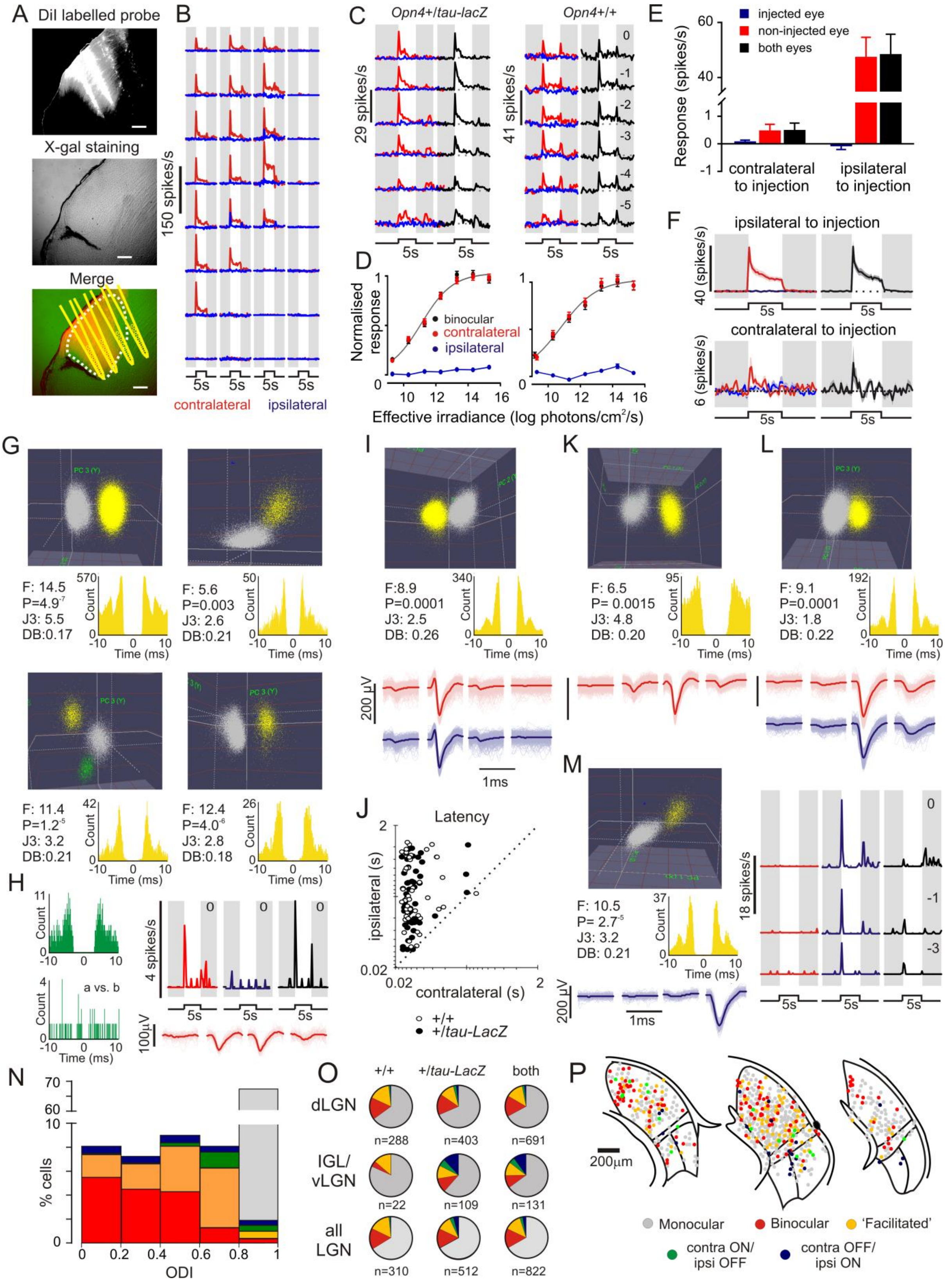
A DiI labelled probe
X-gal staining
Merge
B
150 spikes/s
5s
contralateral
ipsilateral
C
Opn4+/tau-lacZ
Opn4+/+
29 spikes/s
41 spikes/s
0
-1
-2
-3
-4
-5
D
Normalised response
binocular
contralateral
ipsilateral
Effective irradiance (log photons/cm²/s)
E
Response (spikes/s)
injected eye
non-injected eye
both eyes
contralateral to injection
ipsilateral to injection
F
ipsilateral to injection
contralateral to injection
40 (spikes/s)
6 (spikes/s)
G
F: 14.5
P=4.9⁻⁷
J3: 5.5
DB:0.17
F: 5.6
P=0.003
J3: 2.6
DB:0.21
F: 11.4
P=1.2⁻⁵
J3: 3.2
DB:0.21
F: 12.4
P=4.0⁻⁶
J3: 2.8
DB:0.18
Count
Time (ms)
H
a vs. b
4 spikes/s
100µV
I
F:8.9
P=0.0001
J3: 2.5
DB: 0.26
200 µV
1ms
J
Latency
ipsilateral (s)
contralateral (s)
+/+
+/tau-LacZ
K
F: 6.5
P= 0.0015
J3: 4.8
DB: 0.20
L
F: 9.1
P=0.0001
J3: 1.8
DB: 0.22
M
F: 10.5
P= 2.7⁻⁵
J3: 3.2
DB: 0.21
18 spikes/s
N
% cells
ODI
O
+/+
+/tau-LacZ
both
dLGN
IGL/ vLGN
all LGN
n=288
n=403
n=691
n=22
n=109
n=131
n=310
n=512
n=822
P
200µm
Monocular
Binocular
'Facilitated'
contra ON/ ipsi OFF
contra OFF/ ipsi ON

Figure S1. Anatomical and functional mapping of LGN eye-specific responses. Related to Figure 1. (A) Fluorescence microscopy for DiI-labelled electrode tracks (top), light microscopy for X-gal staining of the IGL in *Opn4*$^{+/tau\text{-}lacZ}$ mice (middle panel) and pseudocolored merged image (bottom) showing projected recording site locations across ventromedial dLGN. Medial electrode tracks in A were more clearly visualised in the adjacent section (not shown). Scale bars = 200µm. (B) Example multiunit firing responses detected at the electrode positions indicated in A following 5s light steps (410nm; 14.4 log effective photons/cm^2/s) restricted to ipsi- or contralateral eye. Note the sharp cut-off in multiunit firing at electrodes located outside the LGN boundaries. (C) Responses of a monocular LGN neuron from *Opn4*$^{+/tau\text{-}lacZ}$ (left) and *Opn4*$^{+/+}$ mice (right). Numbers above traces indicate log intensity relative to the maximum (15.4 log photons/cm^2/s). (D) Mean±SEM normalised firing responses (0-500ms after light step) of 343 monocular *Opn4*$^{+/tau\text{-}lacZ}$ neurons (left) and 205 *Opn4*$^{+/+}$ cells (right) plotted as a function of stimulus irradiance (4-parameter sigmoid fit). Responses to ipsilateral stimulation are absent and fit coefficients for binocular and contralateral stimuli were statistically indistinguishable (F-test; both $P>0.05$). There was no significant difference in relative sensitivity between *Opn4*$^{+/tau\text{-}lacZ}$ and *Opn4*$^{+/+}$ responses (F-tests; $P>0.05$). (E-F) Mean±SEM multiunit firing response (5s step; 15.4 log effective photons/cm^2/s) at LGN electrodes following unilateral intravitreal TTX injection (1mM; 1µl volume). Note complete lack of LGN response to stimulating injected eye either contralateral or ipsilateral to injection (data from 45 and 34 recording sites respectively; n=2 bilateral recordings). (G) Principle component feature space, sort quality statistics and autocorrelograms for Binocular units in Figure 1A (yellow clusters; grey clusters represent unsorted waveforms that crossed threshold at that recording site). Sort statistics include MANOVA F and P values and J3 and Davies-Bouldin (DB) metrics. High J3 and low DB indicative of compact well separated clusters. (H) In one case (lower left panel of G) a second monocular unit was isolated at the same electrode site (green cluster). Auto/crosscorrelograms for this cell (left), spike response to 5s light steps (right; top) and evoked spike waveforms (bottom). (I-K) Principle component feature space and sort details for Facilitated and Antagonistic cells in Figure 1F,I and L respectively. Conventions as in G. (L) Latency to response onset for contra- vs. ipsilateral evoked responses of 107 facilitated cells (57 *Opn4*$^{+/tau\text{-}lacZ}$; 50 $^{+/+}$ littermates). (M) Additional example of an Antagonistic binocular cell where inhibitory component is only visible under binocular stimulation due to low basal firing (representative of 3 ipsilateral-ON and 1 contralateral-ON cell). (N) Ocular dominance index (ODI) for all cells reported in Figure 1 (low values correspond to equally matched binocular responses, 1=monocular). (O) Proportions of visually responsive cells exhibiting monocular and binocular responses, split according to LGN subdivision and/or genotype. (P) Estimated anatomical locations of cells used to generate smoothed cell density maps in Figure 1E.

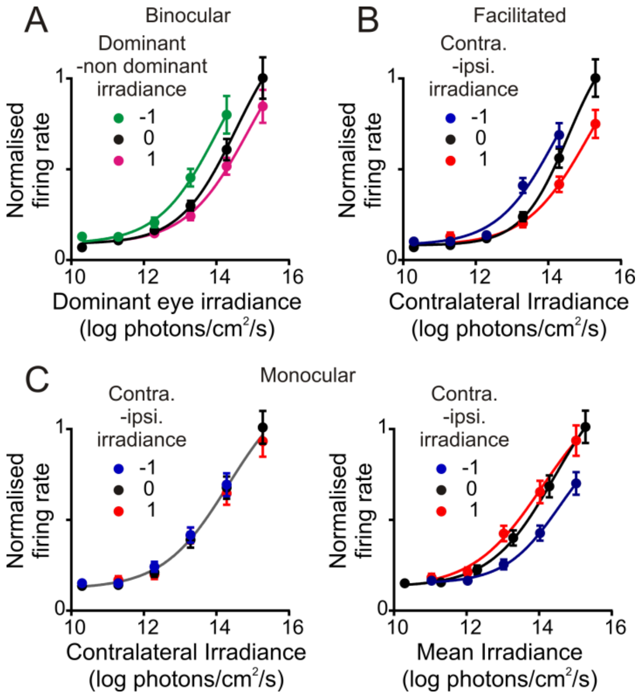

Figure S2. Irradiance coding properties of LGN cells. Related to Figure 2. (A-B) Normalised steady state firing (mean±SEM) of irradiance coding Binocular cells and Facilitated cells (n=57 and 84 respectively) re-plotted from Figure 2E and F but here quantified solely according to irradiance at dominant/contralateral eye. Note that, expressed in this way, the relationships vary according to the difference in interocular irradiance (sigmoid fit coefficients significantly different; F-test: P<0.001 for both). (C) Normalised steady state firing (mean±SEM) of irradiance coding monocular cells (n=175). Data can be fit with a single sigmoid curve when quantified in terms of irradiance at the contralateral eye, regardless of interocular difference in irradiance (left; F-test; P=0.98). The same is not true when these data are re-plotted according to mean binocular irradiance (right; F-test; P<0.001).

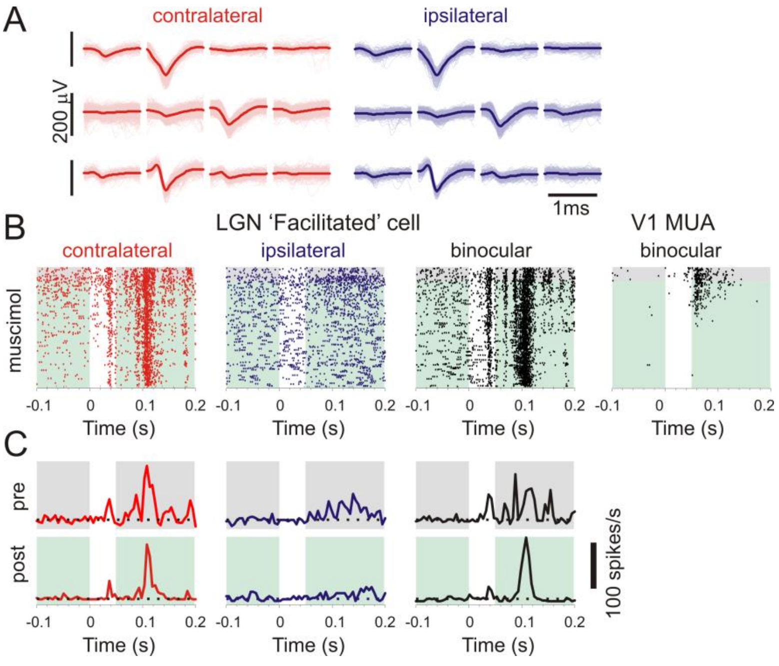

Figure S3. **Binocular and Facilitated cell responses during cortical inactivation.** Related to Figure 3. (A) Spike waveforms evoked by ipsilateral and contralateral flashes for the units in Figure 3A. (B) Peristimulus raster for an LGN Facilitated cell response to flashes (50ms; 15.4 log photons/cm^2/s) targeting one or both eyes. Stimuli run 5min before to 45min after cortical inactivation by topical application of 1mM muscimol. Far right panels representative of simultaneously recorded V1 multiunit response. (C) Peristimulus spike rate histograms (5ms bins) for responses shown in B, evoked over the 5min epoch before muscimol application and 40-45min after drug application. Note the weak/imprecise ipsilateral response disappears after cortical inactivation.

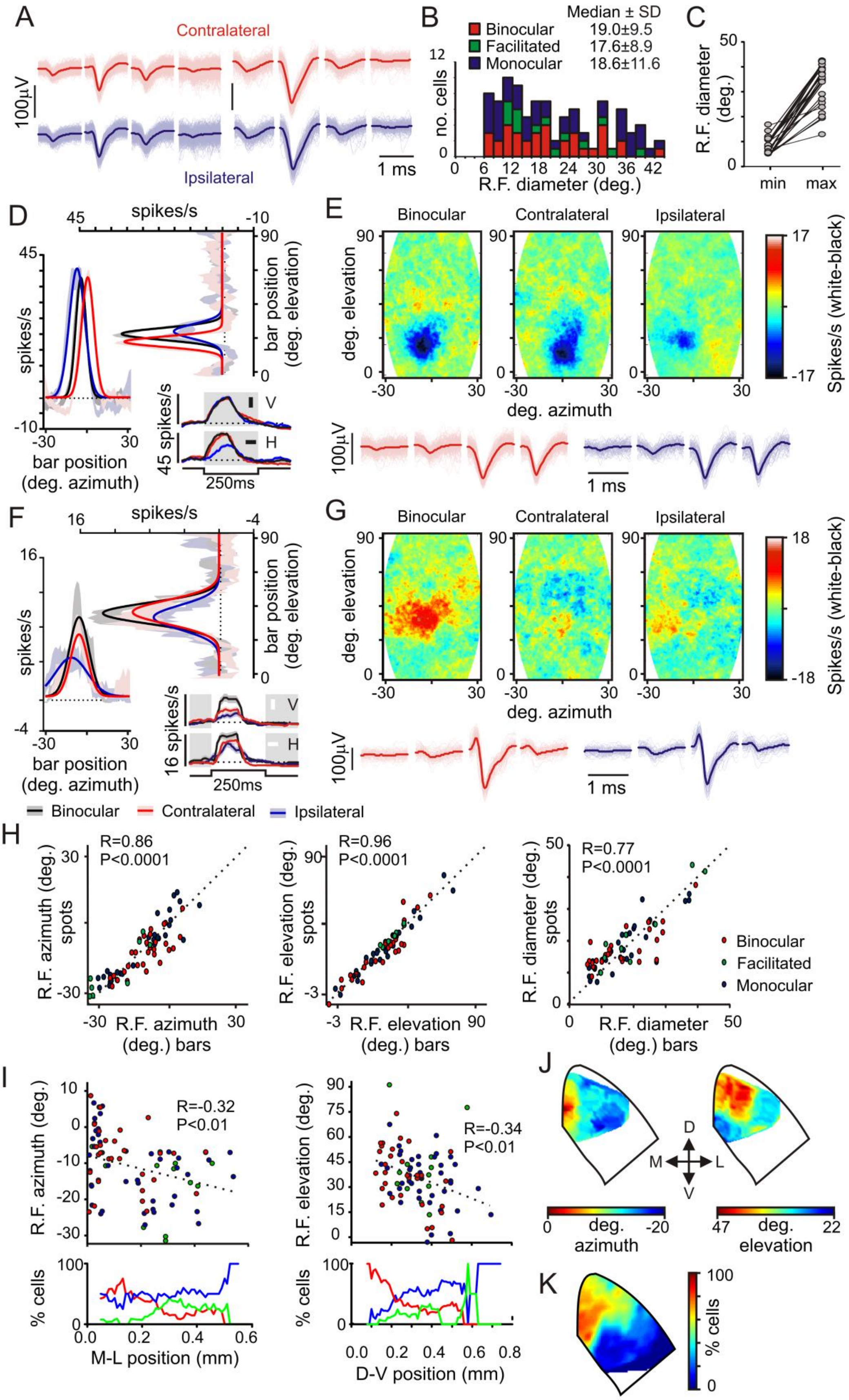

A
Contralateral
Ipsilateral
100µV
1 ms
B
Median ± SD
Binocular 19.0±9.5
Facilitated 17.6±8.9
Monocular 18.6±11.6
no. cells
R.F. diameter (deg.)
C
R.F. diameter (deg.)
min
max
D
spikes/s
bar position (deg. elevation)
bar position (deg. azimuth)
45 spikes/s
250ms
V
H
E
Binocular
Contralateral
Ipsilateral
deg. elevation
deg. azimuth
Spikes/s (white-black)
100µV
1 ms
F
spikes/s
bar position (deg. elevation)
bar position (deg. azimuth)
16 spikes/s
250ms
G
Binocular
Contralateral
Ipsilateral
deg. elevation
deg. azimuth
Spikes/s (white-black)
Binocular
Contralateral
Ipsilateral
H
R=0.86
P<0.0001
R.F. azimuth (deg.) spots
R.F. azimuth (deg.) bars
R=0.96
P<0.0001
R.F. elevation (deg.) spots
R.F. elevation (deg.) bars
R=0.77
P<0.0001
R.F. diameter (deg.) spots
R.F. diameter (deg.) bars
Binocular
Facilitated
Monocular
I
R.F. azimuth (deg.)
R=-0.32
P<0.01
% cells
M-L position (mm)
R.F. elevation (deg.)
R=-0.34
P<0.01
% cells
D-V position (mm)
J
D
M
L
V
deg. azimuth
deg. elevation
K
% cells

Figure S4. **Spatial and retinotopic organisation of receptive fields within the LGN binocular segment.** Related to Figure 4. (A) Spike waveforms evoked by ipsi- and contralateral stimuli for units in Figure 4A. (B) Histogram of receptive field (RF) diameters under binocular viewing for the various classes of LGN neurons. (C) Plot of minimum vs, maximum RF diameter among simultaneously recorded cells (n=23 viewing conditions with 3 or more simultaneously recorded cells). (D) RF maps for a Binocular cell with OFF responses to flashing horizontal and vertical bars under either monocular or binocular viewing conditions. Solid lines indicate Gaussian fit, shaded areas indicate mean+SEM firing response (8 trials), angles expressed relative to skull midpoint. Inset indicates mean±SEM response to optimal horizontal or vertical black bars (10ms bins, 100ms boxcar smoothing). Dotted line indicates mean baseline firing rate (0-125ms before bar appearance). (E) Sparse noise RF map for unit in D plotting difference in spikes evoked by white vs. black spots. Tangent correction is applied to calculation of azimuth angles. Note the good correspondence between the two independent measures of RF properties. Bottom traces represent spike waveforms evoked by ipsi- and contralateral stimuli. (F-G) RF properties of another Binocular cell to flashing bars (F) and sparse noise (G), conventions as in D and E. Note, in this cell, monocular components become hard to quantify under sparse noise mapping. (H) RF correspondence for 30/37 cells mapped with both bars and sparse noise (n=15 Binocular, 4 Facilitated and 11 monocular cells under one or more viewing conditions). (I) Retinotopic organisation of RFs mapped in the dLGN using flashing bar stimuli (under binocular viewing conditions, n=35 Binocular cells, 14 Facilitated cells and 45 monocular cells). Coordinates are expressed relative to the medial and dorsal borders of the LGN. Bottom plots show relative proportion of each cell type as a function of anatomical position (calculated using 100µm moving window). (J) Colour maps of data from I, showing mean azimuth and elevation as a function of anatomical location within the dLGN. (K) Percentage of all cells tested (n=235) responding to flashing bar stimuli presented within the central binocular zone. Note the high proportion of dorsomedially located dLGN cells responding and low proportions in the ventrolateral (monocular) region.

Supplemental Experimental Procedures

Animals

All animal use was in accordance with the Animals, Scientific Procedures, Act of 1986 (UK) and received institutional ethics committee and UK Home Office approval. *Opn4*$^{+/tau\text{-}lacZ}$ mice [S1] and wildtype (*Opn4*$^{+/+}$) littermates were housed under a 12-hour dark/light cycle environment at a temperature of 22°C with food and water ad libitum.

In Vivo Neurophysiology

Urethane (1.55g/kg) anaesthetised adult male mice (50-100 days) were prepared for stereotaxic surgery and insertion of multielectrode arrays as described previously [S2]. Recording probes (A4X8-5mm-50-200-177; Neuronexus, MI, USA) consisting of 4 shanks (spaced 200µm), each with 8 recordings sites (spaced 50µm) were coated with fluorescent dye (CM-DiI; Invitrogen, Paisley, UK) and then inserted either unilaterally (n=16) or bilaterally (n=16) into the LGN. Depending on the LGN region targeted and the number of electrodes employed, in most cases probes were inserted at angles 14-20° from vertical on the medial to lateral plane (sometimes 0°). Probes were initially lowered to a depth 100µm above the target based on stereotaxic coordinates [S3] and then probed with a series of 10x2s binocular test flashes. Based on the distribution of electrode sites exhibiting light-evoked multiunit activity and the orientation of the electrode array relative to the optic tract, probes were then lowered up to 200µm, in order to maximise the number of channels in the LGN. Aside from this initial positioning no further attempt was made to adjust probe position to detect any particular kind of visual response. Accordingly, we consider the distribution of cell types encountered in this study to be an essentially unbiased assessment. In some experiments (n=7) one 4x8 probe was inserted vertically in sagittal orientation to target the mediodorsal aspect of the dLGN (2.1mm lateral to the midline) and a second probe 12° from vertical in sagittal orientation to target deep layers of the V1 binocular zone (recording sites spanning depths between 500-850µm).

After allowing 30min for neural activity to stabilise following probe insertion, wideband neural signals were acquired using a Recorder64 system (Plexon, TX, USA), amplified (x2000) and digitized at 40kHz. Action potentials were discriminated from these signals offline as 'virtual'-tetrode waveforms (Figure 1A; Figure S1) using custom MATLAB scripts (Mathworks, MA, USA). In brief, data were high pass filtered in forward and reverse directions (300Hz, 4th order Butterworth) and grouped as overlapping sets of linear tetrodes (3 tetrodes covering the 8 recording sites on each shank). Tetrode waveforms (40 samples/ch.) were then discriminated by threshold crossing (typically 45-55µV) and sorted manually using commercial principle components based software (Offline sorter, Plexon, TX, USA). Single unit isolation was confirmed by reference to MANOVA F statistics, J3 and Davies-Bouldin validity metrics (Offline sorter) and the presence of a distinct refractory period (>1.5ms) in the interspike interval distribution. Special care was taken to ensure that no cell was discriminated more than once on overlapping tetrodes (confirmed via crosscorrelogram analysis of unit firing).

Pharmacological manipulations

For experiments involving cortical inactivation, a large craniotomy (~1mm diam.) was made over V1 and muscimol (1mM in 0.9% saline; Sigma, Dorset, UK) was applied topically to cover the exposed cortical surface. Over the 1-2h following drug application, we occasionally re-applied the drug to ensure constant coverage. Visually evoked responses were monitored throughout across deep cortical layers (as described above), to ensure the effectiveness of this manipulation.

For retinal inactivation, 1mM tetrodotoxin (Tocris, Bristol, UK) was injected intravitreally (under microscope visualisation) into the left eye (1µl total volume) using a 34 gauge

Hamiliton RN needle with extra fine tip (Esslab, Essex, UK). Immediately following injection electrodes were lowered bilaterally into the LGN and recording proceeded as described above.

Visual Stimuli & Analysis
All light measurements were performed using a calibrated spectroradiometer (Bentham instruments, Reading, UK).

Full Field Stimuli
Full field visual stimuli were generated via two LEDs (λmax 410nm; half-width: ±7nm; Thorlabs, NJ, USA) independently controlled via LabVIEW (National Instruments, TX, USA) and neutral density filter wheels (Thorlabs). Light was supplied to the subject via 7mm diameter flexible fibre optic light guides (Edmund Optics; York, UK), positioned 5mm from each eye and enclosed within internally reflective plastic cones that fit snugly over each eye – preventing any off-target effects due to scattered light.

Responses to such stimuli were assessed using two different protocols. The first was designed to determine the relative magnitude and sensitivity of eye specific responses in LGN neurons. Here mice were maintained in darkness and 5s light steps were applied in an interleaved fashion to contra- and/or ipsilateral eyes for a total of 10 repeats at logarithmically increasing intensities spanning 9.8-15.8 log photons/cm^2/s (interstimulus interval 20-50s depending on intensity).

Our choice of 410nm LED stimuli was based on the fact that all mouse photoreceptors display similar sensitivity in this part of the spectrum [S4]. After correction for pre-receptoral filtering [S5], effective photon fluxes for each mouse opsin were between 0.5 (M- and S-cone opsins) and 0.3 log units (melanopsin) dimmer than the values reported above. Owing of these properties, our measures of sensitivity are not biased towards responses originating from any specific photoreceptor(s). In particular, the dorsal-ventral gradient in cone-opsin expression in the mouse retina [S6, S7] should have no impact on our measures of relative sensitivity. Intensities reported in the manuscript reflect effective irradiance for rod opsin, which is intermediate between cones and melanopsin (9.4-15.4 log photons/cm^2/s).

For latency measurements we calculated peristimulus histograms for the above stimuli (1ms bin size, Gaussian smoothing: σ = 5ms) and found the first bin that exceed the 95% confidence limits of the prestimulus (0-1s) firing activity. Values reported in the text represent the fastest response observed (usually evoked at the highest intensity tested). Estimates of ocular dominance index (ODI) were based on that used previously [S8]; (Dominant – Non-dominant)/(Dominant+Non-dominant). To account for responses Antagonistic cells, here we used the absolute response amplitude. Hence, for all cells, low ODI values correspond to well-matched binocular responses.

For experiments involving cortical inactivation, we also applied a series of interleaved 50ms flashes (effective irradiance 15.4 log photons/cm^2/s; ISI=1s) to one or both eyes for an epoch starting 5min before drug application and lasting at least 50min. To investigate more sustained response components we subsequently repeated a series of 10X5s light steps at full irradiance as described above.

The second protocol we employed was designed to dissociate response components dependent on stimulus brightness vs. stimulus contrast (i.e. absolute vs. relative light intensity). Here we stepped light intensity independently at each eye every 5s in a pseudorandom sequence spanning effective irradiances between 10.4-15.4 log photons/cm^2/s (total number of steps =840). The sequence was generated such that, at any one time, the difference in intensity between the two eyes was no more than 2 decimal units and the instantaneous step in light intensity at each eye was one of five possible values (±2,

1 or 0 log units). To determine contrast-dependent components we then averaged cellular responses (0-500ms post change in light intensity) as a function of step magnitude at either eye across all irradiances investigated. Data reported in the manuscript are restricted to steps providing contrast at only one eye or equal contrast at both eyes.

To assess LGN response components that tracked stimulus brightness, we reanalysed the above to extract steady state firing (1s epochs occurring at least 4s after step in light intensity) as a function of absolute irradiance at either eye (independently or in combination). For each cell, we then calculated Shannon mutual information between steady state firing (discretised to 6 levels) and monocular/binocular irradiance using standard formulae [S9]. These values were compared to mutual information estimates obtained for that cell following shuffling of the stimulus sequence (1000 repeats). A cell was considered to encode irradiance when monocular/binocular information calculated in such a manner exceeded the 95th percentile of the distribution obtained following shuffling. For cell populations encoding significant information about stimulus irradiance we then extracted steady-state firing activity observed at varying binocular or monocular irradiances and plotted these as a function of the difference in irradiance between the two eyes. For this analysis we only included data where the differences in binocular irradiance was ≤1 log unit, since the stimulus design was such that greater differences were statistically more likely to have immediately followed large changes in light intensity.

Spatially Structured Stimuli
The full field stimuli described above have the advantage that, in principle, they allow for stimulation of all LGN neurons, regardless of which part of the retina they receive input from. A potential downside is that, since LGN neurons typically exhibit stimulus size selectivity [S10, S11], they will not evoke optimal responses from all cells. Our analysis indicates this is unlikely to have significantly skewed our estimates of relative cell density: the vast majority of dLGN units we recorded responded robustly to full field stimuli, with only 35/726 cells that did not exhibit measureable responses. A somewhat higher proportion of non-responsive cells were found in the IGL/vLGN region (38/169 cells, predominantly those located around the medial portion of the vLGN). Most importantly, we did not find any cells (from 18 multielectrode placements) that responded to spatially structured but not full field stimuli (including 16 of the above-mentioned unresponsive dLGN cells).

For receptive field mapping, stimuli were delivered via an LCD display (width: 26.8cm height: 47.4cm; Hanns-G HE225DPB) angled at 45° from vertical and placed directly in front of the animal such that the screen centre was 15cm anterior and 15cm superior to the midpoint between the eyes. Under these conditions we calculated that the horizontal and vertical meridians of stimulus display subtended 63.4° azimuth and 96° elevation respectively. Based on previous analyses of binocular visual space in the mouse [S7], we estimate this display occupied ~70% of the binocular zone with minimal intrusion into monocular visual space.

We did not attempt to map receptive fields outside of the central binocular zone. We assume that the majority of cells unresponsive to our spatial stimuli (primarily monocular) had receptive fields outside the region covered by our display. While this assumption was born out by our analysis of retinotopic organisation (Figure S4I-K), it is also possible that a small proportion of these cells were instead tuned to stimulus features that we did not investigate (e.g. [S11]).

Stimuli were generated and controlled via MATLAB using the Psychophysics toolbox [S12, S13] and comprised white or black flashing bars (430 and 3.3 sc. cd/m^2 respectively) superimposed on a background of the opposite polarity. The screen was divided into a 45x80 grid and vertical or horizontal bars (occupying 5 adjacent grid squares; ~7° at

horizontal and vertical meridians) appeared at each possible location (in random sequence) for 250ms followed by a blank screen for 250ms. Thus there was considerable spatial overlap between possible bar positions. These stimuli were run for a total of 8 repeats sequentially for each bar orientation, polarity and screen location, under conditions with one or both eyes viewing. Receptive field parameters were then determined separately for azimuth and elevation under all conditions by plotting the mean response to stimuli of appropriate polarity (100ms epochs starting 35-125ms after bar appearance) as a function of bar position. These were then fit with 1-D Gaussians to estimate receptive field centre position and diameter (full width at half maximum). Values for diameter and response amplitude reported in the text represent the averages of estimates obtained using vertical and horizontal bars of the appropriate polarity. For calculations of visual angle, tangent correction was applied in the azimuthal direction only.

In some experiments we also employed a second set of sparse noise stimuli based on those described previously [S11]. These comprised white and black spots presented on a grey background at a density such that on average 16% of the screen area was covered at any one time. Spots were 2,4,8,16 and 32° in diameter and presented at a density inversely proportional to their size such that, on average, each spot size occupied an equal fraction of the visual display. Under each viewing condition, 10 min sequences of these stimuli were applied at 4 frames/s. Data were analysed by reverse correlation separately for black and white spots (to avoid averaging out ON/OFF responses of nonlinear cells); for each pixel of the screen we calculated the mean firing rate over the 250ms frame duration (50ms epochs) as a function of pixel intensity and subtracted the mean firing rates when that pixel was not covered by a spot. Receptive fields were then calculated by fitting 2-D Gaussians to the largest absolute magnitude response across the two resulting spatiotemporal response maps.

Using these sparse noise stimuli, we were able to map RFs for most (30/37) cells that also responded to flashing bars (Figure S4D-H; no cells responded to the sparse noise stimuli but not to bars). We suspect that the lack of any quantifiable RF in the remaining cells (which all had relatively weak responses to flashing bars) reflects the lower effective contrast of the sparse noise stimuli (spots presented on a grey background) vs. our previous approach (bars presented on a black or white background). Similarly, of 15 Binocular cells that responded to sparse noise stimuli under binocular viewing conditions, 3 failed to exhibit quantifiable RFs under at least one of the monocular conditions (in each case where the response to bars was weak; Figure S4F,G). These 3 cells each exhibited substantially larger responses when stimuli were presented to both eyes than under monocular viewing conditions.

Despite this slightly lower efficiency of the sparse noise approach, where we could map RFs with both techniques we observed a good correlation in terms of RF position and diameter (Figure S4H). Thus, although the relationship between RF diameters observed under the two approaches was not perfect (Figure S4H), there was no systematic difference between the two approaches (mean difference: 1.1±0.6°; paired t-test; $P>0.05$).

To probe for more sluggish changes in cell firing evoked by stimuli in binocular visual space we also presented low frequency (0.2Hz) reversing gratings. These consisted of horizontally orientated black and white bars of various spatial frequencies (~0.2,0.1,0.08 and 0.04 cycles/°, + full screen flashes) and were delivered, as above, under both binocular and monocular viewing conditions.

To ensure truly monocular presentation of all stimuli above, where appropriate, irradiance at the non-stimulated eye was held constant (effective irradiance: 14 log photons/cm^2/s) using the same apparatus used to deliver full field stimuli. Background irradiance in the

experimental room was 13.6 log effective photons/cm^2/s (based on rod sensitivity; M-opsin = 13.7, S-opsin = 11.8, Melanopsin 13.5 log effective photons/cm^2/s).

Consistent with previous reports that eye-movements are minimal in anaesthetised mice [S10], these are extremely unlikely to have had a significant influence on the estimates of spatial response properties reported here. Specifically, we assessed this possibility by looking for coordinated changes (mean absolute difference) in receptive field position and size among simultaneously recorded cells. Firstly we compared these properties as determined by our responses to flashing bars under monocular and binocular viewing conditions (mapping sessions occurring 45-90min apart). To avoid confounding effects of binocular interactions, we restricted this analysis to monocular cells and found evidence of only very minimal changes in eye position (azimuth: 1.5±0.3; elevation: 2.0±0.5; diameter: 1.6±0.4; mean±SEM from 8 viewing conditions with 4 or more monocular cells). Secondly, we looked for coordinated changes in properties measured using bars vs. sparse noise (mapping sessions ~20min apart). Here we included all responsive cells regardless of functional class and found similarly minimal differences (azimuth: 2.1±0.5; elevation: 1.3±0.3; diameter: 1.7±0.4; mean±SEM from 9 viewing conditions with 4 or more cells). See also analysis in Figure S4C, indicating that we detect both large and small RFs among simultaneously recorded cells.

Histology
At the end of each experiment, mice were perfused transcardially with 0.1M phosphate buffered saline followed by 4% paraformaldehyde. The brain was removed and post-fixed in 4% paraformaldehyde for 30min and subsequently cryoprotected in 30% sucrose. The following day, brains were sectioned at 100µm on a freezing sledge microtome and either mounted directly onto slides (wildtype mice) using Vectasheild (Vectorlaboratories Ltd., Peterbourogh, UK) or first processed for X-gal staining (*Opn4*$^{+/tau\text{-}lacZ}$) as described below.

X-gal staining was performed as previously described [S1]. Brain sections were washed twice for 10 minutes each in buffer B (0.1M PBS at pH 7.4, 2mM $MgCl_2$, 0.01% Na-desoxycholate and 0.02% IGEPAL). Sections were then incubated for 18 hours in staining solution [buffer B with potassium ferricyanide (5mM), potassium ferrocyanide (5mM) and X-gal (Bioline Reagents Ltd, UK; 1mg/ml)] at 37°C in darkness. Following staining, sections were washed twice for 5 minutes in 0.1M PBS and mounted to slides using Vectashield as above.

After mounting DiI-labelled probe placements were visualised under a fluorescent microscope (Olympus BX51) with appropriate filter sets and, where appropriate, X-gal staining was visualised by standard light microscopy. Resulting images were then scaled to account for shrinkage (based on the known distance between electrode shanks) and aligned with appropriate stereotaxic atlas figures [S3] using the position and orientation of the optic tract and IGL (as revealed by X-gal staining) as landmarks. Anatomical locations of recorded cells were then estimated from these images, based on the known geometry of the electrode array. Consistent with previous modelling work [S14], we think it very unlikely that any isolated unit was located more than 50µm away from the recording site where we observe the largest spike amplitudes. Hence, we never observed single unit spikes that crossed our spike detection threshold on more than two channels of the 50µm spaced recording array. Similarly, we always observed a very sharp cut-off in visually evoked multiunit firing at electrode sites estimated (from our dye labelling) to lie outside the borders of the LGN (Figure S1A-B).

To produce maps of cell type densities, we first assigned cells to one of 3 anatomical templates corresponding to rostral, mid or caudal LGN. The small number of visually unresponsive cells we encountered were excluded this analysis. We then calculated the relative proportion of each visually responsive cell type using a moving circular window

(radius 100µm; step size 50µm) and smoothed the resulting maps by cubic spline interpolation. A similar procedure was used to produce maps of LGN retinotopic organisation except in this case, to increase sampling coverage, we mapped positions onto a single LGN template (correcting anatomical coordinates relative to the medial and dorsal borders of the LGN for each probe placement).

Supplemental References

S1. Hattar, S., Kumar, M., Park, A., Tong, P., Tung, J., Yau, K.W., and Berson, D.M. (2006). Central projections of melanopsin-expressing retinal ganglion cells in the mouse. J Comp Neurol *497*, 326-349.

S2. Brown, T.M., Gias, C., Hatori, M., Keding, S.R., Semo, M., Coffey, P.J., Gigg, J., Piggins, H.D., Panda, S., and Lucas, R.J. (2010). Melanopsin contributions to irradiance coding in the thalamo-cortical visual system. PLoS Biol *8*, e1000558.

S3. Paxinos, G.F., K.B.J. (2001). The mouse brain in stereotaxic coordinates: Second Edition, (San Diego: Academic Press).

S4. Govardovskii, V.I., Fyhrquist, N., Reuter, T., Kuzmin, D.G., and Donner, K. (2000). In search of the visual pigment template. Vis Neurosci *17*, 509-528.

S5. Jacobs, G.H., and Williams, G.A. (2007). Contributions of the mouse UV photopigment to the ERG and to vision. Doc Ophthalmol *115*, 137-144.

S6. Applebury, M.L., Antoch, M.P., Baxter, L.C., Chun, L.L., Falk, J.D., Farhangfar, F., Kage, K., Krzystolik, M.G., Lyass, L.A., and Robbins, J.T. (2000). The murine cone photoreceptor: a single cone type expresses both S and M opsins with retinal spatial patterning. Neuron *27*, 513-523.

S7. Sterratt, D.C., Lyngholm, D., Willshaw, D.J., and Thompson, I.D. (2013). Standard anatomical and visual space for the mouse retina: computational reconstruction and transformation of flattened retinae with the Retistruct package. PLoS Comput Biol *9*, e1002921.

S8. Zhao, X., Liu, M., and Cang, J. (2013). Sublinear binocular integration preserves orientation selectivity in mouse visual cortex. Nat Commun *4*, 2088.

S9. Montemurro, M.A., Senatore, R., and Panzeri, S. (2007). Tight data-robust bounds to mutual information combining shuffling and model selection techniques. Neural Comput *19*, 2913-2957.

S10. Grubb, M.S., and Thompson, I.D. (2003). Quantitative characterization of visual response properties in the mouse dorsal lateral geniculate nucleus. J Neurophysiol *90*, 3594-3607.

S11. Piscopo, D.M., El-Danaf, R.N., Huberman, A.D., and Niell, C.M. (2013). Diverse visual features encoded in mouse lateral geniculate nucleus. J Neurosci *33*, 4642-4656.

S12. Brainard, D.H. (1997). The Psychophysics Toolbox. Spat Vis *10*, 433-436.

S13. Pelli, D.G. (1997). The VideoToolbox software for visual psychophysics: transforming numbers into movies. Spat Vis *10*, 437-442.

S14. Pettersen, K.H., and Einevoll, G.T. (2008). Amplitude variability and extracellular low-pass filtering of neuronal spikes. Biophys J *94*, 784-802.
