# Supplementary material for: Binocular Integration in the Mouse Lateral Geniculate Nuclei
Source: Curr Biol. 2014 Jun 2;24(11):1241–7. doi: 10.1016/j.cub.2014.04.014 (PMC4046226; doi:10.1016/j.cub.2014.04.014)
Supplement: Document S2. Article plus Supplemental Information [file mmc2.pdf]

Current Biology 24, 1241–1247, June 2, 2014 ©2014 The Authors http://dx.doi.org/10.1016/j.cub.2014.04.014

Report

Binocular Integration in the Mouse Lateral Geniculate Nuclei

Michael Howarth,[1] Lauren Walmsley,[1]
and Timothy M. Brown[1,*]
[1]Faculty of Life Sciences, University of Manchester,
Manchester M13 9PT, UK

Summary

A key task for the visual system is to combine spatially overlapping representations of the environment, viewed by either eye, into a coherent image. In cats and primates, this is accomplished in the cortex [1], with retinal outputs maintained as separate monocular maps en route through the lateral geniculate nucleus (LGN). While this arrangement is also believed to apply to rodents [2, 3], this has not been functionally confirmed. Accordingly, here we used multielectrode recordings to survey eye-specific visual responses across the mouse LGN. Surprisingly, while we find that regions of space visible to both eyes do indeed form part of a monocular representation of the contralateral visual field, we find no evidence for a corresponding ipsilateral representation. Instead, we find many cells that can be driven via either eye. These inputs combine to enhance the detection of weak stimuli, forming a binocular representation of frontal visual space. This extensive thalamic integration marks a fundamental distinction in mechanisms of binocular processing between mice and other mammals.

Results

Binocular Response Types in the Mouse LGN

To assess eye-specific inputs to the visual thalamus we performed unilateral/bilateral multielectrode recordings across the lateral geniculate nucleus (LGN) of anesthetized mice (n = 39; 55 electrode placements). Based upon published anatomy [2, 3], we expected to find individual neurons responsive to either the contralateral or ipsilateral eye in the mouse dorsal LGN (dLGN; primary thalamocortical relay). We further aimed to determine whether a similar arrangement applies to LGN regions important for accessory visual function (intergeniculate leaflet and ventral part [IGL/vLGN]; [4, 5]).

To help distinguish between dLGN and IGL/vLGN recordings, we performed a subset of these (n = 27; 36 electrode placements) in *Opn4*$^{+/tau\text{-}lacZ}$ mice [6, 7] in which the IGL is readily detected by β-galactosidase staining (Figure S1A and S1B available online). Additional recordings from wild-type littermates (n = 19 placements in 12 mice) produced essentially identical results (Figures 1B, 1C, S1C, and S1D). Accordingly, we have combined these data sets for subsequent analysis.

We first recorded LGN responses to full-field stimuli (410 nm light-emitting diode; 5 s) applied at varying intensity to one or both eyes. As expected, most visually responsive LGN cells (n = 548/822) were driven exclusively by the contralateral retina (Figurea S1C, S1D, and S1N–S1P). Surprisingly, however, none of the remaining cells exhibited purely ipsilateral visual responses. Instead these displayed varying types of binocular interaction, of which the most common form (n = 127/274) was a brisk increase in firing elicited by stimulation of either eye (Figure 1A). These "binocular" cells were found at highest density around zones of anatomically defined ipsilateral retinal projections (Figures 1E and S1P; mediodorsal portion of the dLGN and the IGL/vLGN).

The relative magnitude of responses to stimulation of either eye varied for binocular cells (Figures 1A and 1B), but latencies were typically very similar, suggesting a direct convergence of ipsilateral/contralateral retinal ganglion cells (RGCs) onto the same LGN neuron. The monocular components were also markedly subadditive (Figures 1C and 1D), such that there was no clear response enhancement on binocular stimulation.

These properties rule out two trivial interpretations of our data. First, subadditive binocular responses exclude inadequate unit isolation (in which case the responses should be approximately additive; see also Figures 1A and S1G). Second, we didn't observe the large differences in response threshold (Figures 1B and 1D) expected if one of the components resulted from insufficient optical isolation of our stimuli. To further rule out a contribution of off-target effects, we performed additional LGN recordings after retinal inactivation by intravitreal tetrodotoxin injection (Figures S1E and S1F). In no case did we observe any response appearing to originate from the injected eye.

We also observed many LGN neurons (n = 107) with contralateral-driven responses whose activity was modulated by bright stimuli presented to the ipsilateral eye (Figures 1F–1H). In these "facilitated" cells (most commonly found ventrolaterally in the dLGN; Figure 1E), monocular responses partially summed, leading to enhanced steady state firing upon binocular stimulation. Here, ipsilateral responses exhibited unusually sluggish kinetics (Figure S1L), suggesting that they were not directly driven by RGC inputs.

We also observed cells with "antagonistic" binocular responses (Figures 1I–1N), i.e., contralateral ON/ipsilateral OFF (n = 18) or the converse (n = 22). In a few cases, the inhibitory (OFF) component was not visible under monocular stimulation, but only as a dramatically reduced ON response under binocular stimulation (Figure S1M; n = 4). These antagonistic cells were only found at high density within the IGL/vLGN region (Figure 1E), forming a very small proportion of the dLGN neurons sampled (Figures S1O and S1P; ~3% of the total). We have thus focused the remainder of our analysis on binocular and facilitated cells.

Contrast and Irradiance Coding Properties

Since we hypothesized that the activity of binocular and facilitated cells might differentially depend on the absolute versus relative brightness of our stimuli (irradiance versus contrast), we next employed a protocol that allowed us to dissociate components of the response dependent on these features (see the Supplemental Experimental Procedures).

We were particularly interested to understand the properties of binocular cells. We reasoned that the lack of binocular summation after light steps applied from darkness may have resulted from the unusually high contrast of these stimuli.

*Correspondence: timothy.brown@manchester.ac.uk
This is an open access article under the CC BY license (http://creativecommons.org/licenses/by/3.0/).

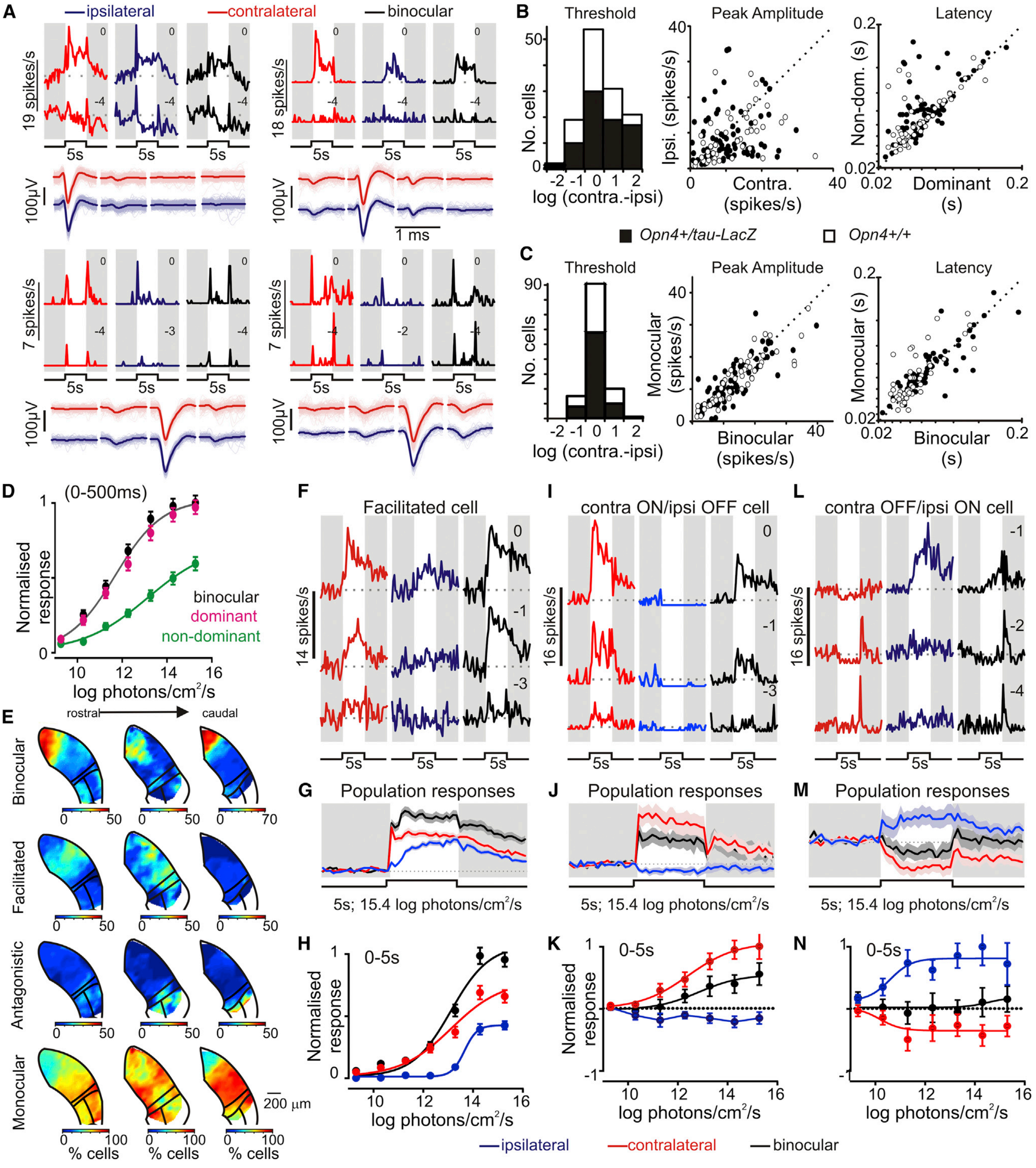

Figure 1. Binocular Visual Responses in Mouse LGN Neurons

(A) Threshold and peak responses (means of ten trials) of four binocular LGN neurons to 5 s light steps applied to one or both eyes (410 nm light-emitting diode; numbers above traces indicate log intensity relative to the maximum: 15.4 log photons/cm^2/s). Dotted lines in each trace indicate the mean prestimulus firing (0–5 s before step). Bottom traces represent "virtual" tetrode spike waveforms for each unit during stimuli restricted to either the ipsilateral or contralateral eye. Thick line represents the mean spike shape.

(B) Relative log threshold intensity (left), peak amplitude (middle), and latency (right) for contralateral versus ipsilateral evoked responses of 127 binocular neurons (77 *Opn4*$^{+/\text{tau-lacZ}}$ and 50 *Opn4*$^{+/+}$ littermates).

(C) Analysis as above for binocular versus dominant-eye-driven responses.

(D) Mean ± SEM normalized responses (0–500 ms after light step) as a function of stimulus irradiance. Sigmoid fit coefficients for binocular and dominant-eye-only stimuli were statistically indistinguishable (F test, $p = 0.07$).

(legend continued on next page)

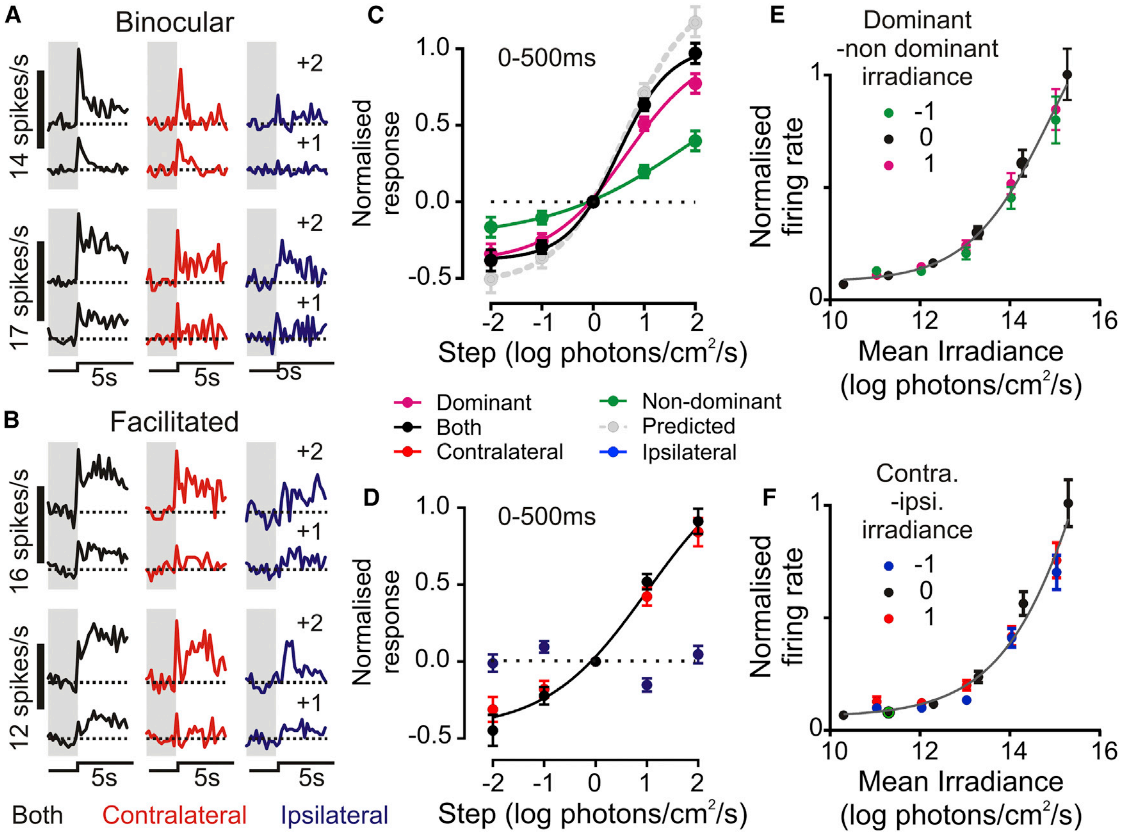

Figure 2. Binocular Input Enhances Visual Coding in LGN Cells

(A and B) Example responses of two binocular (A) and two facilitated (B) cells to 1–2 log unit steps up in intensity targeting one or both eyes (means of 10–60 trials). Dotted lines indicate the mean basal firing rate 0–3 s before step.

(C and D) Mean ± SEM normalized contrast response relationships of binocular (C; n = 97) and facilitated (D; n = 84) cells to stimuli applied to one or both eyes (light-adapted conditions: 11.4–14.4 log photons/cm^2/s). The dotted gray line indicates the linear prediction for the binocular response (contralateral and ipsilateral). To facilitate comparison of cells with different response polarities on the same graph, we sign inverted ON inhibitions and OFF excitations. Binocular cell responses were best fit by separate four-parameter sigmoidal functions (F test, $p < 0.001$). Facilitated cell responses to contralateral and binocular stimuli were not significantly different (F test, $p = 0.29$).

(E and F) Relationship between normalized steady state firing (mean ± SEM) of irradiance coding binocular (E; n = 57) and facilitated (F; n = 84) cells and mean binocular irradiance, plotted according to the difference in interocular irradiance. All data are well described by single four-parameter sigmoid functions regardless of the relative irradiance (F test, p = 0.34 and 0.27 in E and F, respectively).

See also Figure S2 for additional analysis and responses of monocular irradiance coding cells.

To this end, we calculated contrast response relationships for stimuli applied to one or both eyes under light adapted conditions. We found the slope of these curves was indeed significantly steeper under binocular versus dominant-eye-only stimulation (Figures 2A and 2C; F test, $p < 0.05$). Extrapolating from these curves, the step in light intensity evoking a 50% maximal response was reduced from ~11-fold to <5-fold under binocular versus dominant-eye-only stimulation. Thus, at least one function of LGN binocular integration is to enhance detection of weak stimuli.

By distinction, the acute response of facilitated cells was solely determined by the contralateral retina (Figures 2B and 2D); contrast responses were identical under binocular and contralateral-only stimulation (Figure 2D), and ipsilateral responses were essentially absent. An influence of the ipsilateral eye did become visible over later components of the response, however, (Figure 2B) even after modest steps in light intensity.

One interpretation of these data is that the basal activity of facilitated cells provides information about binocular irradiance. Accordingly, we next calculated their steady-state firing as a function of absolute irradiance. Applying an information-theory-based test (see the Supplemental Experimental Procedures), we determined that the basal firing activity of each of these facilitated cells did indeed encode significant ($p < 0.05$) information about binocular irradiance. Accordingly, when

(E) Relative proportion of visually responsive cells showing various binocular or monocular response profiles as a function of anatomical localization within the LGN.

(F–N) response properties of facilitated (F–H) and antagonistic (I–K, contralateral ON/ipsilateral OFF; L–N, contralateral OFF/ipsilateral ON) cells.

(F, I, and L) Example single-cell responses, with conventions as in (A).

(G, J, and M) Mean ± SEM normalized population responses (n = 107, 18, and 22, respectively) to bright light steps targeting one or both eyes.

(H, K, and N) Mean ± SEM normalized irradiance response functions (0–5 s after light step).

See also Figure S1 for further details of unit isolation, spike waveforms, additional data, and anatomical distributions.

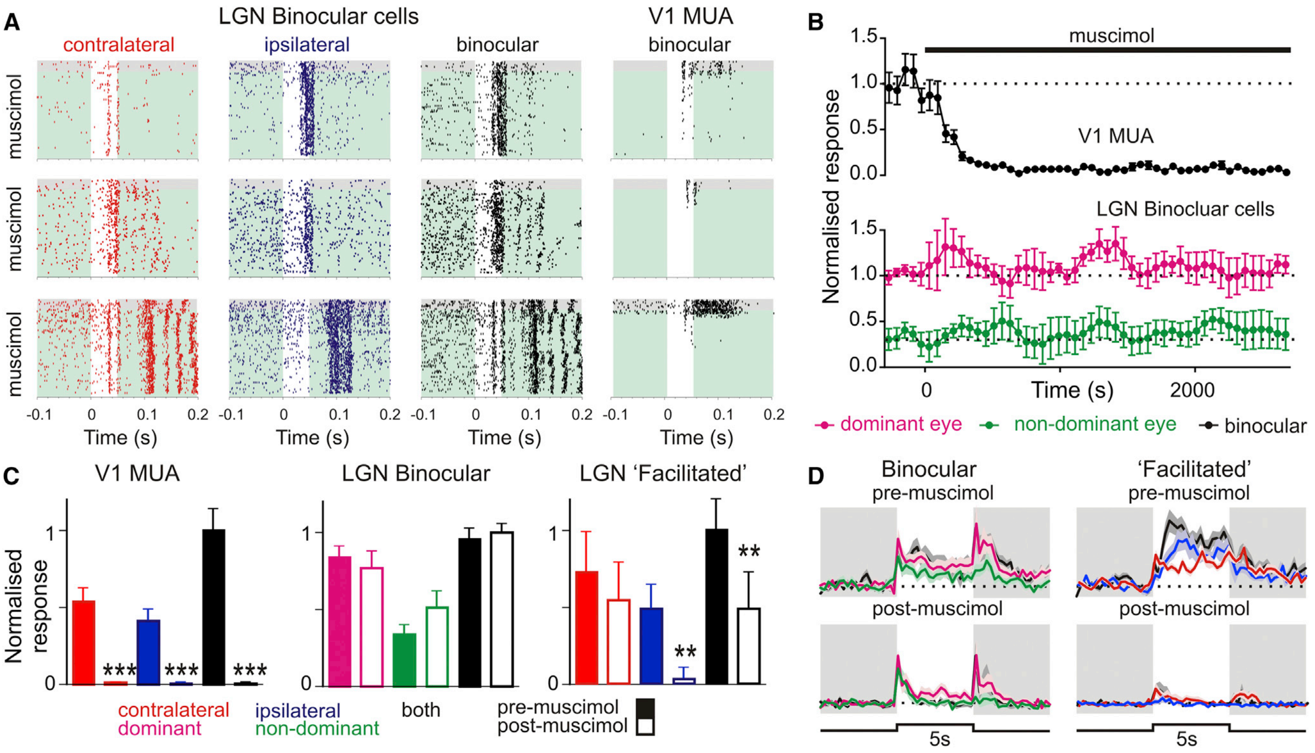

Figure 3. Cortical Feedback Is Not Required for LGN Binocular Cell Responses

(A) Peristimulus rasters for three LGN binocular cell responses to flashes (50 ms; 15.4 log photons/cm^2/s) targeting one or both eyes. Stimuli run from 5 min before to 45 min after cortical inactivation by topical application of 1 mM muscimol. The far-right panels are representative of simultaneously recorded V1 multiunit responses. Note that LGN binocular responses persist long after cortical responses disappear, even when there is a large difference in latency between eye-specific responses (bottom).

(B) Time course of muscimol effects (mean ± SEM normalized response) on V1 multiunit response (n = 110 channels) and LGN binocular cell responses (n = 14).

(C) Mean ± SEM V1 multiunit responses (left; n = 110 channels), LGN binocular cell responses (middle; n = 14), and facilitated cell responses (right; n = 9) across 5 min epochs before muscimol application (filled bars) and 40–60 min after (open bars). Data were analyzed by paired t test (**$p < 0.01$, ***$p < 0.001$).

(D) Mean ± SEM normalized response of binocular (left) and facilitated (right) cells to 5 s light steps (15.4 log photons/cm^2/s) before and after cortical inactivation. The dotted line indicates the mean prestimulus (0–5 s) firing. Note that although transient contrast responses persist, sustained response components are attenuated after muscimol application.

See also Figure S3 for spike waveforms and example facilitated cell responses.

plotted as a function of the average irradiance across the two eyes, steady-state firing was well described by a single sigmoid curve regardless of the interocular difference in brightness (F test, p = 0.27; Figure 3F). The same was not true if instead these data were instead expressed solely in terms of contralateral irradiance (Figure S2B).

This irradiance coding property also extended to many binocular cells (n = 57/91). Activity of this subset of cells was also well described by a single curve when expressed in terms of mean binocular irradiance (Figure 2E; F test, p = 0.34), but not when quantified in terms of irradiance at the dominant eye (Figure S2A). These properties were not a general feature of irradiance coding LGN neurons, however, since we identified many monocular LGN cells (n = 175/363) that solely encoded contralateral irradiance (Figure S2C).

Role of Cortical Feedback

In addition to direct retinal input, a major source of excitatory input to the dLGN comes via feedback from the visual cortex [8, 9]. To establish whether corticothalamic feedback contributed to binocular responses in the LGN, in some experiments (n = 7) we assessed LGN visually evoked activity (50 ms flashes; 15.4 log photons/cm^2/s) after cortical inactivation by topical application of muscimol (GABA$_A$ agonist). As previously reported [10], muscimol produced a profound and persistent inhibition of cortical activity, essentially abolishing firing by 40–60 min (Figures 3A–3C; 99.1% ± 0.7% block). Responses of simultaneously recorded LGN binocular cells remained unchanged, however (Figures 3A–3C; n = 14). This was even true of one cell (Figure 3A) in which ipsilateral responses were significantly slower than those driven by contralateral stimulation.

By contrast, all ipsilateral components of facilitated cell responses disappeared after cortical inactivation (n = 9; Figures 3C and S3B). A further effect was revealed when we then switched to longer (5 s) light steps (45–90 min postmuscimol). While contralateral stimuli still evoked transient increases in firing, all sustained components of the responses of facilitated cells were completely abolished (Figure 3D). We also observed a reduction in sustained responses of binocular cells (Figure 3D), suggesting that the binocular irradiance coding properties described above may rely on cortical feedback.

Spatial Response Properties

Our data above suggest that binocular integration enhances LGN visual coding. One condition of our interpretation is

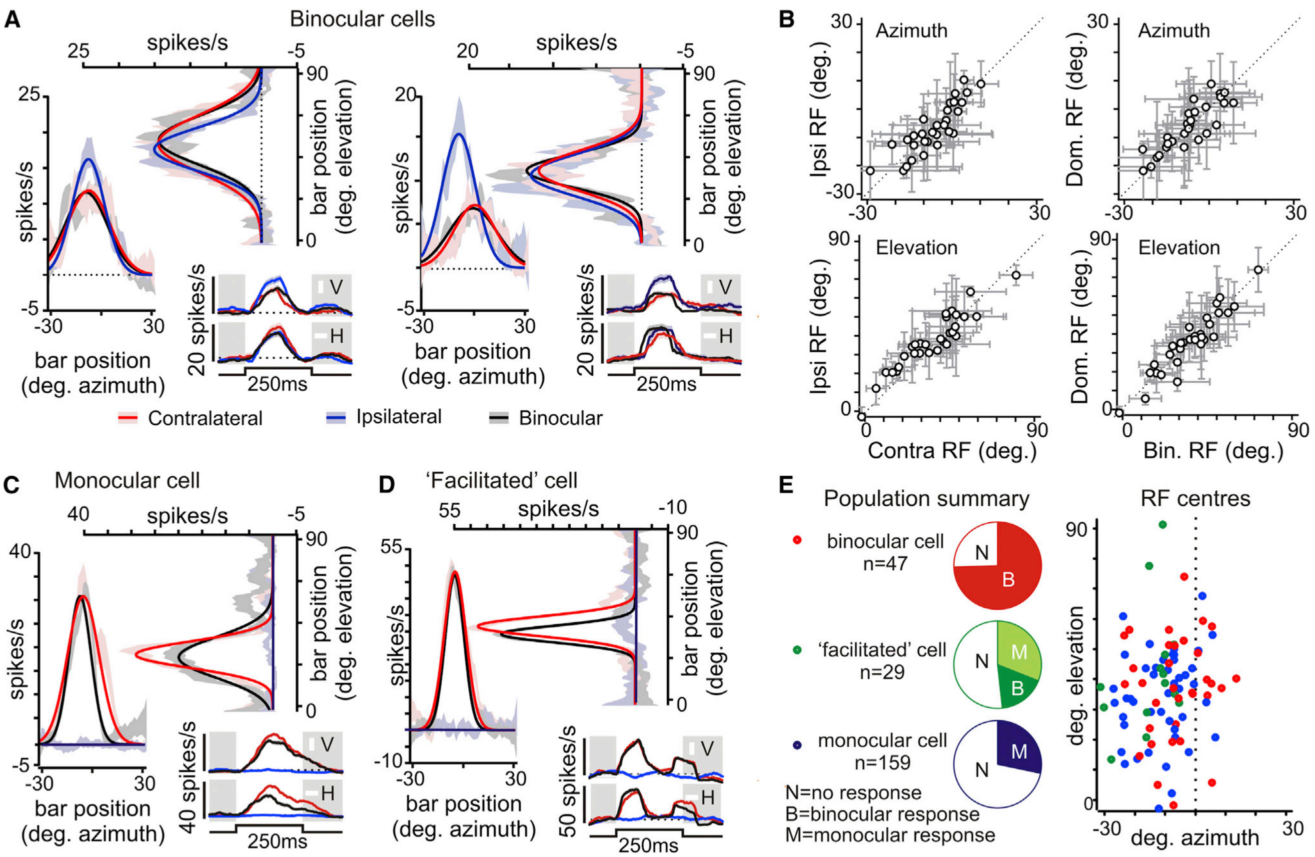

Figure 4. LGN subpopulations encode binocular or monocular representations of frontal visual space.

(A) RF maps for two binocular neurons obtained using flashing horizontal and vertical white bars under either monocular or binocular viewing conditions. Solid lines indicate Gaussian fit, shaded areas indicate mean ± SEM firing response (eight trials), and angles are expressed relative to the skull's midpoint. The inset shows mean ± SEM response to optimal horizontal or vertical bars (10 ms bins, 100 ms boxcar smoothing). The dotted line indicates the mean baseline firing rate (0–125 ms before bar appearance).

(B) Ipsilateral versus contralateral (left) and dominant-eye versus binocular (right) RF correspondence for 30 binocular cells. Error bars represent the estimated RF radius.

(C and D) RF maps for monocular (C) and facilitated (D) cells, with conventions as in (A).

(E) Summary of population data showing proportion of cells exhibiting binocular/monocular RF properties (left) and RF center positions (right) for all responding neurons (n = 35, 14, and 45 for binocular, facilitated, and monocular cells, respectively).

See also Figure S4 for additional examples, analysis, and retinotopic organization.

that both monocular inputs should originate from equivalent regions of visual space. To confirm this and to rule out the possibility that our use of full-field stimuli biased our recordings toward a subset of LGN neurons (see the Supplemental Experimental Procedures), we also examined spatial response properties in a subset of bilateral recordings (n = 9).

To localize the receptive field (RF) centers for binocular cells, we applied horizontal and vertical bar stimuli via a display centered within frontal visual space. Since this display encompassed >70% of the binocular zone, we predicted that it should be possible to determine RF locations for a similar proportion of binocular cells. Accordingly, with both eyes viewing, this was indeed possible for 35/47 binocular cells (Figures 4A, 4B, and 4E). RFs varied in diameter and spatial location (Figures 4B and S4B) and the majority were of the ON variety (n = 24; Figures 4A and S4F), although we also observed OFF (n = 6; Figure S4D) and ON-OFF (n = 5) responses.

We next determined eye-specific contributions to the binocular RFs. Of the 35 cells with RFs within the region surveyed, 30 also responded when stimulation was restricted to either eye alone (Figures 4A, 4B, S4D, and S4E). The signs (ON or OFF) of these monocular RFs were invariably consistent, while the relative amplitudes varied from cell to cell. We also observed modest differences in RF position and/or diameter (Figure 4B; mean ± SEM: azimuth, 3.8° ± 0.5°; elevation, 5.7° ± 0.5°; width, 5.0° ± 1.5°). However, our estimates of such differences were markedly less than the size of the dominant eye RF (median ± SD: 17.6° ± 8.9°), such that eye-specific RFs occupied overlapping regions of visual space (Figure 4B).

We also observed one cell that responded only upon binocular stimulation, and there were four in which we could only map RFs for one eye (two ipsilateral, two contralateral). Each of these cells exhibited substantially larger responses after binocular stimulation than under monocular viewing (78% ± 22%; paired t test, $p < 0.05$). For the other cells, dominant-eye and binocular responses were of similar magnitude (17.3 ± 2.3 versus 15.2 ± 2.2 spikes/s, respectively; paired t test, $p > 0.05$). Similarly, there was no significant difference in RF size between binocular and monocular viewing conditions (mean ± SEM: 1.0° ± 1.5°; $p > 0.05$).

A smaller proportion of facilitated cells (14/29) had measureable RFs within the area of our visual display (Figures 4E

and S4E), and only five cells showed evidence of a binocular response to flashing bars (weak ipsilateral responses or response to binocular only). The remaining cells exhibited purely contralateral visual responses that were similar under binocular and monocular viewing (Figure 4D). To probe for more sluggish responses, we also employed 0.2 Hz reversing gratings of various spatial frequencies. Since these did not reveal any clear binocular response (data not shown), we conclude that the binocular irradiance coding properties described above may operate on a more global scale than the contrast responses of facilitated cells.

Given the substantial binocular integration that we describe here, one might expect that monocular LGN cells would receive input from regions of space visible to only the contralateral eye. In fact, many monocular cells did respond to stimuli within the binocular zone (45/159; Figures 4C and 4E). None of these, however, responded to stimuli restricted to the ipsilateral retina, nor did we find any difference in response amplitude upon contralateral versus binocular viewing (18.0 ± 1.8 versus 16.8 ± 1.6 spikes/s). Thus, while there is no purely ipsilateral representation of binocular visual space, a contralateral-only representation is present within the mouse LGN.

Consistent with previous reports that RF size in the mouse LGN increases with visual eccentricity [11], RFs of all cell classes categorized here tended toward the larger end (Figure S4B) of those generally reported for LGN neurons (11°–17°; [11, 12]). Our analysis indicates that eye movements are unlikely to have been a significantly factor in our experiments (Figure S4C), nor did this finding appear to be an artifact of our RF mapping approach. Indeed, in some experiments (n = 5), we also employed sparse noise stimuli (similar to those used previously in the mouse [12]), and we found a good correlation to estimates of RF position and diameter obtained with bars (Figures S4D–S4H; see also the Supplemental Experimental Procedures).

Finally, we also confirmed that cells responding to our spatial stimuli exhibited the expected retinotopic location within the dLGN. This was the case for both binocular and monocular cells (Figures S4I–S4K), with more dorsomedially located cells preferring spatial stimuli closer to the midline and at greater elevations, as described previously [12, 13].

Discussion

Unexpectedly, we find no evidence for functional segregation of ipsilateral visual signals in the mouse LGN. Instead, we find many cells exhibiting some form of binocular response. Most commonly, these manifest as rapid excitatory responses to contrast in a specific region of space that can be evoked via either eye independently. Integration of these binocular inputs is markedly subadditive for strong stimuli, but acts to increase contrast sensitivity, enhancing responses to low-contrast elements in the visual scene.

Given the generally near-identical latency of their monocular inputs, the most parsimonious explanation for our data is that many binocular cells receive monosynaptic input from either retina. This arrangement would be in stark contrast with the cat or primate LGN, in which binocular interactions (when present) are believed to arise polysynaptically [14–17]. An earlier study did find evidence for direct binocular integration in the rat LGN [18], however, suggesting this may be a common feature of rodent visual systems.

Direct binocular integration seems surprising, given the anatomical separation of LGN retinal inputs. It is of note, however, that the ipsilateral terminal field is relatively thin across most of the dLGN (~100 μm; [2]) by comparison with the dendritic field of mouse thalamocortical projection neurons (~150 μm; [19]). Accordingly, it would seem that only cells at the very center of the dLGN ipsilateral zone could be isolated from contralateral inputs. Indeed, anatomical studies have identified LGN relay cells whose dendritic fields span eye-specific domains [19].

Nonetheless, one would still expect to find some purely ipsilateral neurons. Although rare (1%–2%), we did find a few dLGN cells that exhibited excitatory responses to stimulation of the ipsilateral retina and inhibitory contralateral responses. We suspect direct RGC input to these cells is purely ipsilateral, with the contralateral inhibition provided via interneurons that span eye-specific domains [20]. We also found LGN binocular cells in which the latency difference between ipsilateral and contralateral responses was sufficiently large to allow for indirect integration. Based on our cortical inactivation experiments, it is unlikely that this could arise through top-down influences. Such integration could occur subcortically, however (e.g., [21]).

There have been few functional investigations of binocular integration in the mouse LGN. Binocular input to lateral margins of the dLGN is found only in immature rodents [22, 23], but these studies did not investigate the medial regions where we find binocular cells. Our findings are apparently at odds, however, with a previous report of exclusively ipsilateral mouse LGN neurons [13]. We suspect that this discrepancy relates to the higher contrast of our stimuli relative to this previous work (70% contrast gratings), which we predict would generally evoke weak responses when presented to the nondominant eye alone. Consistent with this view, another recent study using higher-contrast gratings has reported binocular responses in the mouse LGN [24].

Mouse cortical neurons exhibit varying preference toward either eye, hitherto assumed to derive from differential pooling of eye-specific monocular inputs [25–28]. Although our data indicate that much of this diversity is already present at the level of individual LGN neurons, it is also clear that further integration must occur in the cortex, most notably because dLGN RFs (present study; [10–13]) typically lack the strongly oriented structure of V1 simple cells. We find a purely contralateral representation of frontal visual space in the mouse dLGN, and it seems likely that this is combined with the equivalent binocular representation to generate the distribution of ocular dominance observed in cortical neurons.

In addition to conventional binocular integration, we also observed a cortically imposed binocular irradiance signal across many LGN cells. This signal is independent of the magnitude of eye-specific contrast responses and extends to facilitated cells located outside of what is traditionally considered the dLGN binocular zone. Although we have been unable to determine the spatial extent of this influence within individual neurons, given the anatomical distribution of cells exhibiting this property, we suspect that it operates across a more global spatial scale than the classical RF. Such global irradiance signals might provide important contextual information used at higher stages of the visual pathway, for example to infer surface brightness and/or lightness [29].

We also investigated whether binocular processing differed between LGN subregions serving cortical versus subcortical pathways. In this regard, we found a greatly increased prevalence of antagonistic binocular responses in the IGL/vLGN

region, presumably driven by inhibitory connections within and/or between the nuclei of opposing hemispheres [4, 5]. Insofar as these cells provide information about interocular differences in irradiance, we speculate they may be important for visuomotor control, a key proposed function of the IGL/vLGN.

Together, our data reveal fundamental differences in the processing of eye-specific signals within the mouse LGN relative to other mammals. These findings have important implications for our understanding of ecological and evolutionary aspects of visual system organization and for the use of the mouse as a model to understand human vision. In particular, the widespread integration of eye-specific signals in the mouse LGN should be taken into account when investigating binocular responses further along the visual pathway.

Supplemental Information

Supplemental Information includes four figures and Supplemental Experimental Procedures and can be found with this article online at http://dx.doi.org/10.1016/j.cub.2014.04.014.

Acknowledgments

This work was supported by a BBSRC (UK) David Phillips fellowship to T.M.B. We thank Jasmina Cehajic for assistance with intravitreal injections.

Received: July 24, 2013
Revised: February 13, 2014
Accepted: April 4, 2014
Published: May 22, 2014

References

1. Casagrande, V.A., and Boyd, J.D. (1996). The neural architecture of binocular vision. Eye (Lond.) *10*, 153–160.
2. Coleman, J.E., Law, K., and Bear, M.F. (2009). Anatomical origins of ocular dominance in mouse primary visual cortex. Neuroscience *161*, 561–571.
3. Sterratt, D.C., Lyngholm, D., Willshaw, D.J., and Thompson, I.D. (2013). Standard anatomical and visual space for the mouse retina: computational reconstruction and transformation of flattened retinae with the Retistruct package. PLoS Comput. Biol. *9*, e1002921.
4. Harrington, M.E. (1997). The ventral lateral geniculate nucleus and the intergeniculate leaflet: interrelated structures in the visual and circadian systems. Neurosci. Biobehav. Rev. *21*, 705–727.
5. Morin, L.P., and Allen, C.N. (2006). The circadian visual system, 2005. Brain Res. Brain Res. Rev. *51*, 1–60.
6. Hattar, S., Lucas, R.J., Mrosovsky, N., Thompson, S., Douglas, R.H., Hankins, M.W., Lem, J., Biel, M., Hofmann, F., Foster, R.G., and Yau, K.W. (2003). Melanopsin and rod-cone photoreceptive systems account for all major accessory visual functions in mice. Nature *424*, 76–81.
7. Lucas, R.J., Hattar, S., Takao, M., Berson, D.M., Foster, R.G., and Yau, K.W. (2003). Diminished pupillary light reflex at high irradiances in melanopsin-knockout mice. Science *299*, 245–247.
8. Cudeiro, J., and Sillito, A.M. (2006). Looking back: corticothalamic feedback and early visual processing. Trends Neurosci. *29*, 298–306.
9. Jurgens, C.W., Bell, K.A., McQuiston, A.R., and Guido, W. (2012). Optogenetic stimulation of the corticothalamic pathway affects relay cells and GABAergic neurons differently in the mouse visual thalamus. PLoS ONE *7*, e45717.
10. Scholl, B., Tan, A.Y., Corey, J., and Priebe, N.J. (2013). Emergence of orientation selectivity in the Mammalian visual pathway. J. Neurosci. *33*, 10616–10624.
11. Grubb, M.S., and Thompson, I.D. (2003). Quantitative characterization of visual response properties in the mouse dorsal lateral geniculate nucleus. J. Neurophysiol. *90*, 3594–3607.
12. Piscopo, D.M., El-Danaf, R.N., Huberman, A.D., and Niell, C.M. (2013). Diverse visual features encoded in mouse lateral geniculate nucleus. J. Neurosci. *33*, 4642–4656.
13. Grubb, M.S., Rossi, F.M., Changeux, J.P., and Thompson, I.D. (2003). Abnormal functional organization in the dorsal lateral geniculate nucleus of mice lacking the beta 2 subunit of the nicotinic acetylcholine receptor. Neuron *40*, 1161–1172.
14. Murphy, P.C., and Sillito, A.M. (1989). The binocular input to cells in the feline dorsal lateral geniculate nucleus (dLGN). J. Physiol. *415*, 393–408.
15. Guido, W., Tumosa, N., and Spear, P.D. (1989). Binocular interactions in the cat's dorsal lateral geniculate nucleus. I. Spatial-frequency analysis of responses of X, Y, and W cells to nondominant-eye stimulation. J. Neurophysiol. *62*, 526–543.
16. Marrocco, R.T., and McClurkin, J.W. (1979). Binocular interaction in the lateral geniculate nucleus of the monkey. Brain Res. *168*, 633–637.
17. Cheong, S.K., Tailby, C., Solomon, S.G., and Martin, P.R. (2013). Cortical-like receptive fields in the lateral geniculate nucleus of marmoset monkeys. J. Neurosci. *33*, 6864–6876.
18. Grieve, K.L. (2005). Binocular visual responses in cells of the rat dLGN. J. Physiol. *566*, 119–124.
19. Krahe, T.E., El-Danaf, R.N., Dilger, E.K., Henderson, S.C., and Guido, W. (2011). Morphologically distinct classes of relay cells exhibit regional preferences in the dorsal lateral geniculate nucleus of the mouse. J. Neurosci. *31*, 17437–17448.
20. Seabrook, T.A., Krahe, T.E., Govindaiah, G., and Guido, W. (2013). Interneurons in the mouse visual thalamus maintain a high degree of retinal convergence throughout postnatal development. Neural Dev. *8*, 24.
21. Ackman, J.B., Burbridge, T.J., and Crair, M.C. (2012). Retinal waves coordinate patterned activity throughout the developing visual system. Nature *490*, 219–225.
22. Jaubert-Miazza, L., Green, E., Lo, F.S., Bui, K., Mills, J., and Guido, W. (2005). Structural and functional composition of the developing retinogeniculate pathway in the mouse. Vis. Neurosci. *22*, 661–676.
23. Ziburkus, J., and Guido, W. (2006). Loss of binocular responses and reduced retinal convergence during the period of retinogeniculate axon segregation. J. Neurophysiol. *96*, 2775–2784.
24. Zhao, X., Liu, M., and Cang, J. (2013). Sublinear binocular integration preserves orientation selectivity in mouse visual cortex. Nat. Commun. *4*, 2088.
25. Dräger, U.C. (1978). Observations on monocular deprivation in mice. J. Neurophysiol. *41*, 28–42.
26. Gordon, J.A., and Stryker, M.P. (1996). Experience-dependent plasticity of binocular responses in the primary visual cortex of the mouse. J. Neurosci. *16*, 3274–3286.
27. Scholl, B., Burge, J., and Priebe, N.J. (2013). Binocular integration and disparity selectivity in mouse primary visual cortex. J. Neurophysiol. *109*, 3013–3024.
28. Longordo, F., To, M.S., Ikeda, K., and Stuart, G.J. (2013). Sublinear integration underlies binocular processing in primary visual cortex. Nat. Neurosci. *16*, 714–723.
29. Kingdom, F.A. (2011). Lightness, brightness and transparency: a quarter century of new ideas, captivating demonstrations and unrelenting controversy. Vision Res. *51*, 652–673.

Current Biology, Volume *24*

Supplemental Information

Binocular Integration in the Mouse Lateral Geniculate Nuclei

Michael Howarth, Lauren Walmsley, and Timothy M. Brown

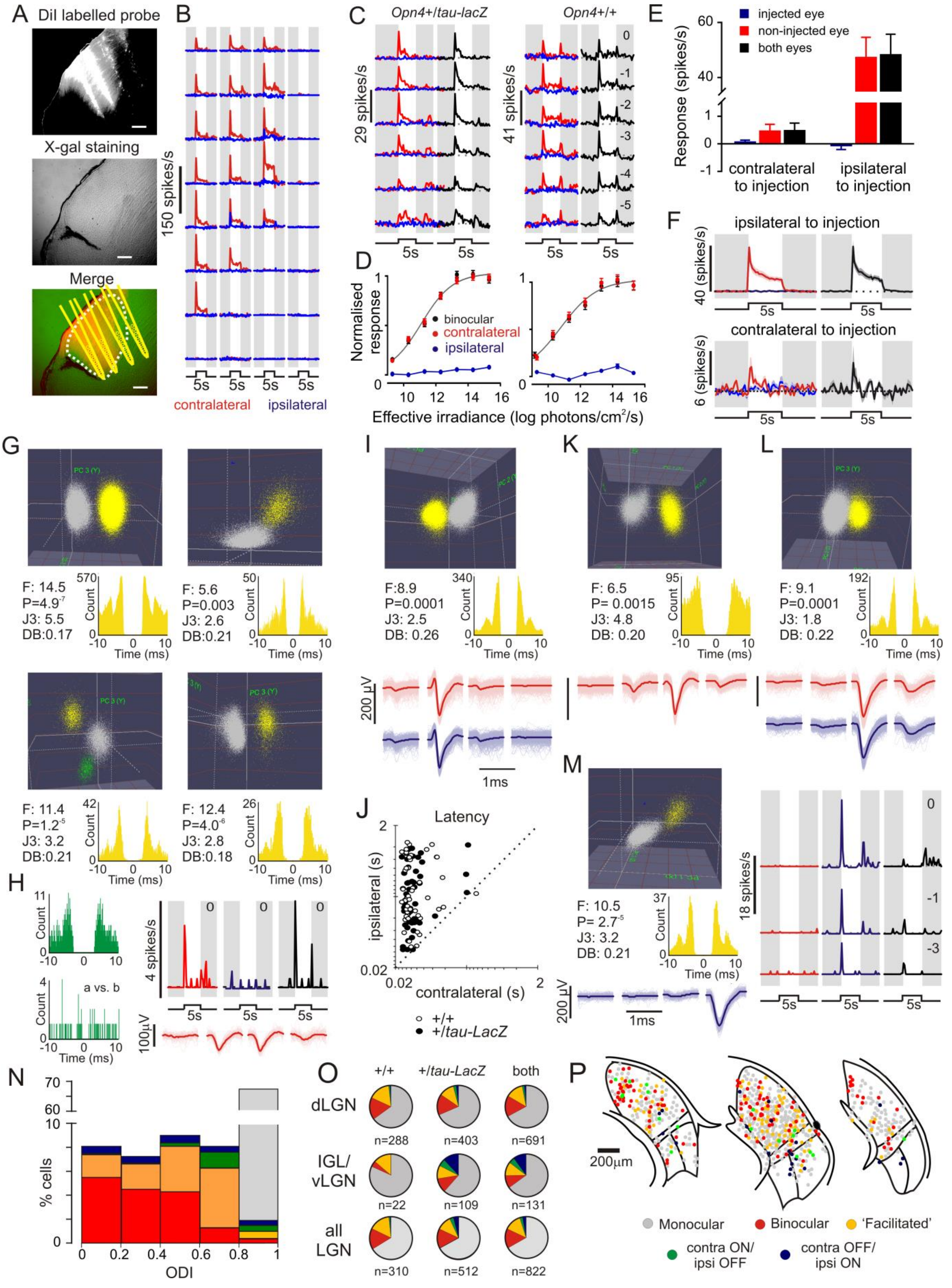

A DiI labelled probe
X-gal staining
Merge
B
150 spikes/s
5s
contralateral
ipsilateral
C
Opn4+/tau-lacZ
Opn4+/+
29 spikes/s
41 spikes/s
0
-1
-2
-3
-4
-5
D
Normalised response
binocular
contralateral
ipsilateral
Effective irradiance (log photons/cm²/s)
E
injected eye
non-injected eye
both eyes
Response (spikes/s)
contralateral to injection
ipsilateral to injection
F
ipsilateral to injection
40 (spikes/s)
contralateral to injection
6 (spikes/s)
G
F: 14.5
P=4.9⁻⁷
J3: 5.5
DB:0.17
F: 5.6
P=0.003
J3: 2.6
DB:0.21
F: 11.4
P=1.2⁻⁵
J3: 3.2
DB:0.21
F: 12.4
P=4.0⁻⁶
J3: 2.8
DB:0.18
Count
Time (ms)
H
a vs. b
4 spikes/s
100µV
I
F:8.9
P=0.0001
J3: 2.5
DB: 0.26
K
F: 6.5
P= 0.0015
J3: 4.8
DB: 0.20
L
F: 9.1
P=0.0001
J3: 1.8
DB: 0.22
200 µV
1ms
J
Latency
ipsilateral (s)
contralateral (s)
+/+
+/tau-LacZ
M
F: 10.5
P= 2.7⁻⁵
J3: 3.2
DB: 0.21
18 spikes/s
N
% cells
ODI
O
+/+
+/tau-LacZ
both
dLGN
IGL/ vLGN
all LGN
n=288
n=403
n=691
n=22
n=109
n=131
n=310
n=512
n=822
P
200µm
Monocular
Binocular
'Facilitated'
contra ON/ ipsi OFF
contra OFF/ ipsi ON

Figure S1. Anatomical and functional mapping of LGN eye-specific responses. Related to Figure 1. (A) Fluorescence microscopy for DiI-labelled electrode tracks (top), light microscopy for X-gal staining of the IGL in *Opn4*$^{+/tau\text{-}lacZ}$ mice (middle panel) and pseudocolored merged image (bottom) showing projected recording site locations across ventromedial dLGN. Medial electrode tracks in A were more clearly visualised in the adjacent section (not shown). Scale bars = 200µm. (B) Example multiunit firing responses detected at the electrode positions indicated in A following 5s light steps (410nm; 14.4 log effective photons/cm^2/s) restricted to ipsi- or contralateral eye. Note the sharp cut-off in multiunit firing at electrodes located outside the LGN boundaries. (C) Responses of a monocular LGN neuron from *Opn4*$^{+/tau\text{-}lacZ}$ (left) and *Opn4*$^{+/+}$ mice (right). Numbers above traces indicate log intensity relative to the maximum (15.4 log photons/cm^2/s). (D) Mean±SEM normalised firing responses (0-500ms after light step) of 343 monocular *Opn4*$^{+/tau\text{-}lacZ}$ neurons (left) and 205 *Opn4*$^{+/+}$ cells (right) plotted as a function of stimulus irradiance (4-parameter sigmoid fit). Responses to ipsilateral stimulation are absent and fit coefficients for binocular and contralateral stimuli were statistically indistinguishable (F-test; both P>0.05). There was no significant difference in relative sensitivity between *Opn4*$^{+/tau\text{-}lacZ}$ and *Opn4*$^{+/+}$ responses (F-tests; P>0.05). (E-F) Mean±SEM multiunit firing response (5s step; 15.4 log effective photons/cm^2/s) at LGN electrodes following unilateral intravitreal TTX injection (1mM; 1µl volume). Note complete lack of LGN response to stimulating injected eye either contralateral or ipsilateral to injection (data from 45 and 34 recording sites respectively; n=2 bilateral recordings). (G) Principle component feature space, sort quality statistics and autocorrelograms for Binocular units in Figure 1A (yellow clusters; grey clusters represent unsorted waveforms that crossed threshold at that recording site). Sort statistics include MANOVA F and P values and J3 and Davies-Bouldin (DB) metrics. High J3 and low DB indicative of compact well separated clusters. (H) In one case (lower left panel of G) a second monocular unit was isolated at the same electrode site (green cluster). Auto/crosscorrelograms for this cell (left), spike response to 5s light steps (right; top) and evoked spike waveforms (bottom). (I-K) Principle component feature space and sort details for Facilitated and Antagonistic cells in Figure 1F,I and L respectively. Conventions as in G. (L) Latency to response onset for contra- vs. ipsilateral evoked responses of 107 facilitated cells (57 *Opn4*$^{+/tau\text{-}lacZ}$; 50 $^{+/+}$ littermates). (M) Additional example of an Antagonistic binocular cell where inhibitory component is only visible under binocular stimulation due to low basal firing (representative of 3 ipsilateral-ON and 1 contralateral-ON cell). (N) Ocular dominance index (ODI) for all cells reported in Figure 1 (low values correspond to equally matched binocular responses, 1=monocular). (O) Proportions of visually responsive cells exhibiting monocular and binocular responses, split according to LGN subdivision and/or genotype. (P) Estimated anatomical locations of cells used to generate smoothed cell density maps in Figure 1E.

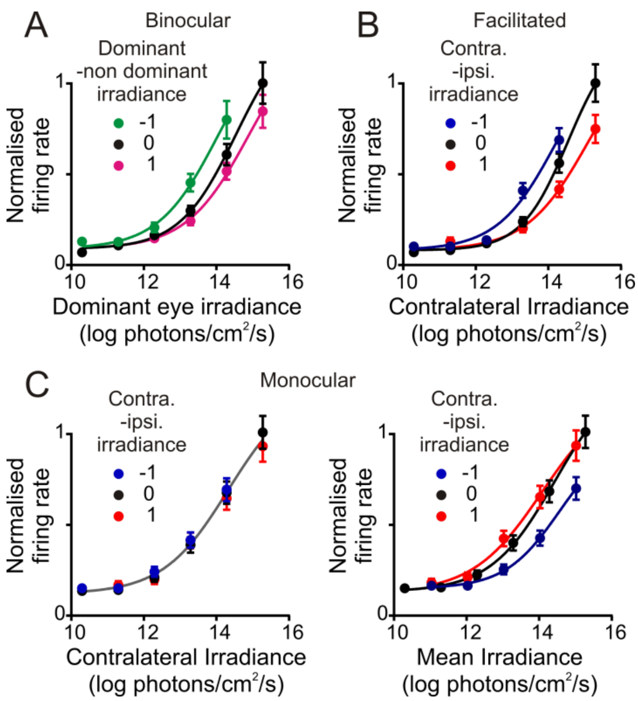

Figure S2. Irradiance coding properties of LGN cells. Related to Figure 2. (A-B) Normalised steady state firing (mean±SEM) of irradiance coding Binocular cells and Facilitated cells (n=57 and 84 respectively) re-plotted from Figure 2E and F but here quantified solely according to irradiance at dominant/contralateral eye. Note that, expressed in this way, the relationships vary according to the difference in interocular irradiance (sigmoid fit coefficients significantly different; F-test: $P<0.001$ for both). (C) Normalised steady state firing (mean±SEM) of irradiance coding monocular cells (n=175). Data can be fit with a single sigmoid curve when quantified in terms of irradiance at the contralateral eye, regardless of interocular difference in irradiance (left; F-test; $P=0.98$). The same is not true when these data are re-plotted according to mean binocular irradiance (right; F-test; $P<0.001$).

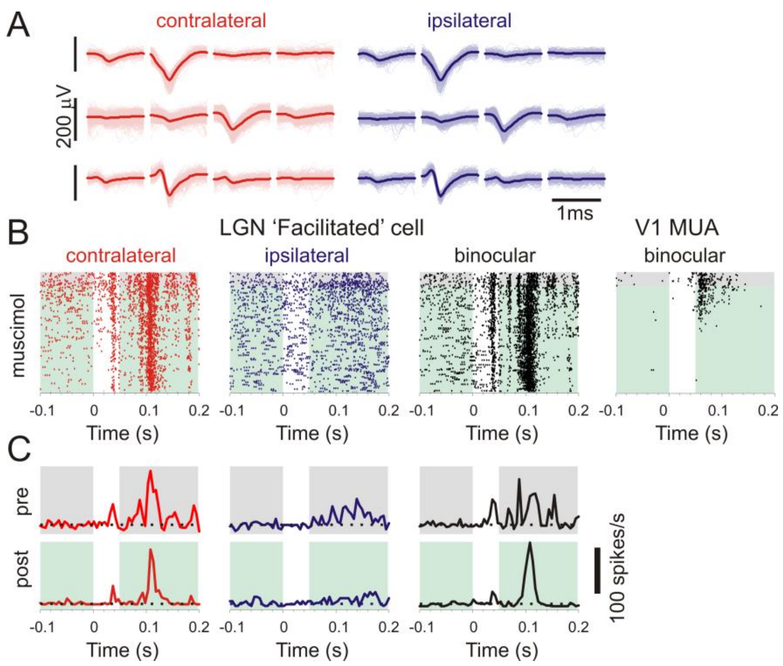

Figure S3. **Binocular and Facilitated cell responses during cortical inactivation.** Related to Figure 3. (A) Spike waveforms evoked by ipsilateral and contralateral flashes for the units in Figure 3A. (B) Peristimulus raster for an LGN Facilitated cell response to flashes (50ms; 15.4 log photons/cm^2/s) targeting one or both eyes. Stimuli run 5min before to 45min after cortical inactivation by topical application of 1mM muscimol. Far right panels representative of simultaneously recorded V1 multiunit response. (C) Peristimulus spike rate histograms (5ms bins) for responses shown in B, evoked over the 5min epoch before muscimol application and 40-45min after drug application. Note the weak/imprecise ipsilateral response disappears after cortical inactivation.

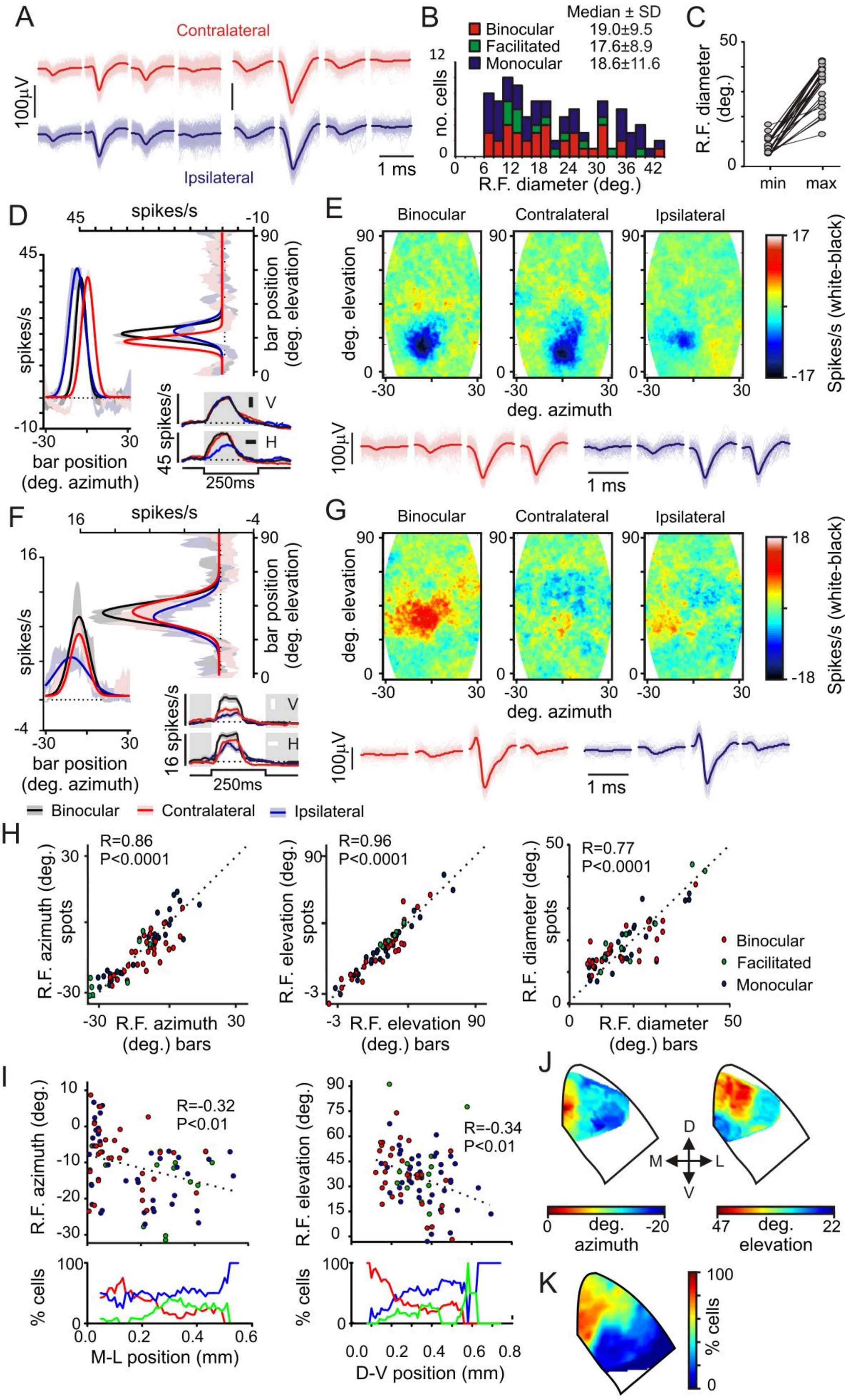

A
Contralateral
Ipsilateral
100µV
1 ms
B
Median ± SD
Binocular 19.0±9.5
Facilitated 17.6±8.9
Monocular 18.6±11.6
no. cells
R.F. diameter (deg.)
C
R.F. diameter (deg.)
min
max
D
spikes/s
bar position (deg. elevation)
bar position (deg. azimuth)
250ms
E
Binocular
Contralateral
Ipsilateral
deg. elevation
deg. azimuth
Spikes/s (white-black)
1 ms
F
spikes/s
bar position (deg. elevation)
bar position (deg. azimuth)
250ms
G
Binocular
Contralateral
Ipsilateral
deg. elevation
deg. azimuth
Spikes/s (white-black)
1 ms
Binocular
Contralateral
Ipsilateral
H
R=0.86
P<0.0001
R.F. azimuth (deg.) spots
R.F. azimuth (deg.) bars
R=0.96
P<0.0001
R.F. elevation (deg.) spots
R.F. elevation (deg.) bars
R=0.77
P<0.0001
R.F. diameter (deg.) spots
R.F. diameter (deg.) bars
Binocular
Facilitated
Monocular
I
R.F. azimuth (deg.)
R=-0.32
P<0.01
R.F. elevation (deg.)
R=-0.34
P<0.01
% cells
M-L position (mm)
D-V position (mm)
J
D
M
L
V
deg. azimuth
deg. elevation
K
% cells

Figure S4. **Spatial and retinotopic organisation of receptive fields within the LGN binocular segment.** Related to Figure 4. (A) Spike waveforms evoked by ipsi- and contralateral stimuli for units in Figure 4A. (B) Histogram of receptive field (RF) diameters under binocular viewing for the various classes of LGN neurons. (C) Plot of minimum vs, maximum RF diameter among simultaneously recorded cells (n=23 viewing conditions with 3 or more simultaneously recorded cells). (D) RF maps for a Binocular cell with OFF responses to flashing horizontal and vertical bars under either monocular or binocular viewing conditions. Solid lines indicate Gaussian fit, shaded areas indicate mean+SEM firing response (8 trials), angles expressed relative to skull midpoint. Inset indicates mean±SEM response to optimal horizontal or vertical black bars (10ms bins, 100ms boxcar smoothing). Dotted line indicates mean baseline firing rate (0-125ms before bar appearance). (E) Sparse noise RF map for unit in D plotting difference in spikes evoked by white vs. black spots. Tangent correction is applied to calculation of azimuth angles. Note the good correspondence between the two independent measures of RF properties. Bottom traces represent spike waveforms evoked by ipsi- and contralateral stimuli. (F-G) RF properties of another Binocular cell to flashing bars (F) and sparse noise (G), conventions as in D and E. Note, in this cell, monocular components become hard to quantify under sparse noise mapping. (H) RF correspondence for 30/37 cells mapped with both bars and sparse noise (n=15 Binocular, 4 Facilitated and 11 monocular cells under one or more viewing conditions). (I) Retinotopic organisation of RFs mapped in the dLGN using flashing bar stimuli (under binocular viewing conditions, n=35 Binocular cells, 14 Facilitated cells and 45 monocular cells). Coordinates are expressed relative to the medial and dorsal borders of the LGN. Bottom plots show relative proportion of each cell type as a function of anatomical position (calculated using 100µm moving window). (J) Colour maps of data from I, showing mean azimuth and elevation as a function of anatomical location within the dLGN. (K) Percentage of all cells tested (n=235) responding to flashing bar stimuli presented within the central binocular zone. Note the high proportion of dorsomedially located dLGN cells responding and low proportions in the ventrolateral (monocular) region.

Supplemental Experimental Procedures

Animals

All animal use was in accordance with the Animals, Scientific Procedures, Act of 1986 (UK) and received institutional ethics committee and UK Home Office approval. *Opn4*$^{+/tau\text{-}lacZ}$ mice [S1] and wildtype (*Opn4*$^{+/+}$) littermates were housed under a 12-hour dark/light cycle environment at a temperature of 22°C with food and water ad libitum.

In Vivo Neurophysiology

Urethane (1.55g/kg) anaesthetised adult male mice (50-100 days) were prepared for stereotaxic surgery and insertion of multielectrode arrays as described previously [S2]. Recording probes (A4X8-5mm-50-200-177; Neuronexus, MI, USA) consisting of 4 shanks (spaced 200µm), each with 8 recordings sites (spaced 50µm) were coated with fluorescent dye (CM-DiI; Invitrogen, Paisley, UK) and then inserted either unilaterally (n=16) or bilaterally (n=16) into the LGN. Depending on the LGN region targeted and the number of electrodes employed, in most cases probes were inserted at angles 14-20° from vertical on the medial to lateral plane (sometimes 0°). Probes were initially lowered to a depth 100µm above the target based on stereotaxic coordinates [S3] and then probed with a series of 10x2s binocular test flashes. Based on the distribution of electrode sites exhibiting light-evoked multiunit activity and the orientation of the electrode array relative to the optic tract, probes were then lowered up to 200µm, in order to maximise the number of channels in the LGN. Aside from this initial positioning no further attempt was made to adjust probe position to detect any particular kind of visual response. Accordingly, we consider the distribution of cell types encountered in this study to be an essentially unbiased assessment. In some experiments (n=7) one 4x8 probe was inserted vertically in sagittal orientation to target the mediodorsal aspect of the dLGN (2.1mm lateral to the midline) and a second probe 12° from vertical in sagittal orientation to target deep layers of the V1 binocular zone (recording sites spanning depths between 500-850µm).

After allowing 30min for neural activity to stabilise following probe insertion, wideband neural signals were acquired using a Recorder64 system (Plexon, TX, USA), amplified (x2000) and digitized at 40kHz. Action potentials were discriminated from these signals offline as 'virtual'-tetrode waveforms (Figure 1A; Figure S1) using custom MATLAB scripts (Mathworks, MA, USA). In brief, data were high pass filtered in forward and reverse directions (300Hz, 4th order Butterworth) and grouped as overlapping sets of linear tetrodes (3 tetrodes covering the 8 recording sites on each shank). Tetrode waveforms (40 samples/ch.) were then discriminated by threshold crossing (typically 45-55µV) and sorted manually using commercial principle components based software (Offline sorter, Plexon, TX, USA). Single unit isolation was confirmed by reference to MANOVA F statistics, J3 and Davies-Bouldin validity metrics (Offline sorter) and the presence of a distinct refractory period (>1.5ms) in the interspike interval distribution. Special care was taken to ensure that no cell was discriminated more than once on overlapping tetrodes (confirmed via crosscorrelogram analysis of unit firing).

Pharmacological manipulations

For experiments involving cortical inactivation, a large craniotomy (~1mm diam.) was made over V1 and muscimol (1mM in 0.9% saline; Sigma, Dorset, UK) was applied topically to cover the exposed cortical surface. Over the 1-2h following drug application, we occasionally re-applied the drug to ensure constant coverage. Visually evoked responses were monitored throughout across deep cortical layers (as described above), to ensure the effectiveness of this manipulation.

For retinal inactivation, 1mM tetrodotoxin (Tocris, Bristol, UK) was injected intravitreally (under microscope visualisation) into the left eye (1µl total volume) using a 34 gauge

Hamiliton RN needle with extra fine tip (Esslab, Essex, UK). Immediately following injection electrodes were lowered bilaterally into the LGN and recording proceeded as described above.

Visual Stimuli & Analysis
All light measurements were performed using a calibrated spectroradiometer (Bentham instruments, Reading, UK).

Full Field Stimuli
Full field visual stimuli were generated via two LEDs (λmax 410nm; half-width: ±7nm; Thorlabs, NJ, USA) independently controlled via LabVIEW (National Instruments, TX, USA) and neutral density filter wheels (Thorlabs). Light was supplied to the subject via 7mm diameter flexible fibre optic light guides (Edmund Optics; York, UK), positioned 5mm from each eye and enclosed within internally reflective plastic cones that fit snugly over each eye – preventing any off-target effects due to scattered light.

Responses to such stimuli were assessed using two different protocols. The first was designed to determine the relative magnitude and sensitivity of eye specific responses in LGN neurons. Here mice were maintained in darkness and 5s light steps were applied in an interleaved fashion to contra- and/or ipsilateral eyes for a total of 10 repeats at logarithmically increasing intensities spanning 9.8-15.8 log photons/cm^2/s (interstimulus interval 20-50s depending on intensity).

Our choice of 410nm LED stimuli was based on the fact that all mouse photoreceptors display similar sensitivity in this part of the spectrum [S4]. After correction for pre-receptoral filtering [S5], effective photon fluxes for each mouse opsin were between 0.5 (M- and S-cone opsins) and 0.3 log units (melanopsin) dimmer than the values reported above. Owing of these properties, our measures of sensitivity are not biased towards responses originating from any specific photoreceptor(s). In particular, the dorsal-ventral gradient in cone-opsin expression in the mouse retina [S6, S7] should have no impact on our measures of relative sensitivity. Intensities reported in the manuscript reflect effective irradiance for rod opsin, which is intermediate between cones and melanopsin (9.4-15.4 log photons/cm^2/s).

For latency measurements we calculated peristimulus histograms for the above stimuli (1ms bin size, Gaussian smoothing: σ = 5ms) and found the first bin that exceed the 95% confidence limits of the prestimulus (0-1s) firing activity. Values reported in the text represent the fastest response observed (usually evoked at the highest intensity tested). Estimates of ocular dominance index (ODI) were based on that used previously [S8]; (Dominant – Non-dominant)/(Dominant+Non-dominant). To account for responses Antagonistic cells, here we used the absolute response amplitude. Hence, for all cells, low ODI values correspond to well-matched binocular responses.

For experiments involving cortical inactivation, we also applied a series of interleaved 50ms flashes (effective irradiance 15.4 log photons/cm^2/s; ISI=1s) to one or both eyes for an epoch starting 5min before drug application and lasting at least 50min. To investigate more sustained response components we subsequently repeated a series of 10X5s light steps at full irradiance as described above.

The second protocol we employed was designed to dissociate response components dependent on stimulus brightness vs. stimulus contrast (i.e. absolute vs. relative light intensity). Here we stepped light intensity independently at each eye every 5s in a pseudorandom sequence spanning effective irradiances between 10.4-15.4 log photons/cm^2/s (total number of steps =840). The sequence was generated such that, at any one time, the difference in intensity between the two eyes was no more than 2 decimal units and the instantaneous step in light intensity at each eye was one of five possible values (±2,

1 or 0 log units). To determine contrast-dependent components we then averaged cellular responses (0-500ms post change in light intensity) as a function of step magnitude at either eye across all irradiances investigated. Data reported in the manuscript are restricted to steps providing contrast at only one eye or equal contrast at both eyes.

To assess LGN response components that tracked stimulus brightness, we reanalysed the above to extract steady state firing (1s epochs occurring at least 4s after step in light intensity) as a function of absolute irradiance at either eye (independently or in combination). For each cell, we then calculated Shannon mutual information between steady state firing (discretised to 6 levels) and monocular/binocular irradiance using standard formulae [S9]. These values were compared to mutual information estimates obtained for that cell following shuffling of the stimulus sequence (1000 repeats). A cell was considered to encode irradiance when monocular/binocular information calculated in such a manner exceeded the 95th percentile of the distribution obtained following shuffling. For cell populations encoding significant information about stimulus irradiance we then extracted steady-state firing activity observed at varying binocular or monocular irradiances and plotted these as a function of the difference in irradiance between the two eyes. For this analysis we only included data where the differences in binocular irradiance was ≤1 log unit, since the stimulus design was such that greater differences were statistically more likely to have immediately followed large changes in light intensity.

Spatially Structured Stimuli

The full field stimuli described above have the advantage that, in principle, they allow for stimulation of all LGN neurons, regardless of which part of the retina they receive input from. A potential downside is that, since LGN neurons typically exhibit stimulus size selectivity [S10, S11], they will not evoke optimal responses from all cells. Our analysis indicates this is unlikely to have significantly skewed our estimates of relative cell density: the vast majority of dLGN units we recorded responded robustly to full field stimuli, with only 35/726 cells that did not exhibit measureable responses. A somewhat higher proportion of non-responsive cells were found in the IGL/vLGN region (38/169 cells, predominantly those located around the medial portion of the vLGN). Most importantly, we did not find any cells (from 18 multielectrode placements) that responded to spatially structured but not full field stimuli (including 16 of the above-mentioned unresponsive dLGN cells).

For receptive field mapping, stimuli were delivered via an LCD display (width: 26.8cm height: 47.4cm; Hanns-G HE225DPB) angled at 45° from vertical and placed directly in front of the animal such that the screen centre was 15cm anterior and 15cm superior to the midpoint between the eyes. Under these conditions we calculated that the horizontal and vertical meridians of stimulus display subtended 63.4° azimuth and 96° elevation respectively. Based on previous analyses of binocular visual space in the mouse [S7], we estimate this display occupied ~70% of the binocular zone with minimal intrusion into monocular visual space.

We did not attempt to map receptive fields outside of the central binocular zone. We assume that the majority of cells unresponsive to our spatial stimuli (primarily monocular) had receptive fields outside the region covered by our display. While this assumption was born out by our analysis of retinotopic organisation (Figure S4I-K), it is also possible that a small proportion of these cells were instead tuned to stimulus features that we did not investigate (e.g. [S11]).

Stimuli were generated and controlled via MATLAB using the Psychophysics toolbox [S12, S13] and comprised white or black flashing bars (430 and 3.3 sc. cd/m^2 respectively) superimposed on a background of the opposite polarity. The screen was divided into a 45x80 grid and vertical or horizontal bars (occupying 5 adjacent grid squares; ~7° at

horizontal and vertical meridians) appeared at each possible location (in random sequence) for 250ms followed by a blank screen for 250ms. Thus there was considerable spatial overlap between possible bar positions. These stimuli were run for a total of 8 repeats sequentially for each bar orientation, polarity and screen location, under conditions with one or both eyes viewing. Receptive field parameters were then determined separately for azimuth and elevation under all conditions by plotting the mean response to stimuli of appropriate polarity (100ms epochs starting 35-125ms after bar appearance) as a function of bar position. These were then fit with 1-D Gaussians to estimate receptive field centre position and diameter (full width at half maximum). Values for diameter and response amplitude reported in the text represent the averages of estimates obtained using vertical and horizontal bars of the appropriate polarity. For calculations of visual angle, tangent correction was applied in the azimuthal direction only.

In some experiments we also employed a second set of sparse noise stimuli based on those described previously [S11]. These comprised white and black spots presented on a grey background at a density such that on average 16% of the screen area was covered at any one time. Spots were 2,4,8,16 and 32° in diameter and presented at a density inversely proportional to their size such that, on average, each spot size occupied an equal fraction of the visual display. Under each viewing condition, 10 min sequences of these stimuli were applied at 4 frames/s. Data were analysed by reverse correlation separately for black and white spots (to avoid averaging out ON/OFF responses of nonlinear cells); for each pixel of the screen we calculated the mean firing rate over the 250ms frame duration (50ms epochs) as a function of pixel intensity and subtracted the mean firing rates when that pixel was not covered by a spot. Receptive fields were then calculated by fitting 2-D Gaussians to the largest absolute magnitude response across the two resulting spatiotemporal response maps.

Using these sparse noise stimuli, we were able to map RFs for most (30/37) cells that also responded to flashing bars (Figure S4D-H; no cells responded to the sparse noise stimuli but not to bars). We suspect that the lack of any quantifiable RF in the remaining cells (which all had relatively weak responses to flashing bars) reflects the lower effective contrast of the sparse noise stimuli (spots presented on a grey background) vs. our previous approach (bars presented on a black or white background). Similarly, of 15 Binocular cells that responded to sparse noise stimuli under binocular viewing conditions, 3 failed to exhibit quantifiable RFs under at least one of the monocular conditions (in each case where the response to bars was weak; Figure S4F,G). These 3 cells each exhibited substantially larger responses when stimuli were presented to both eyes than under monocular viewing conditions.

Despite this slightly lower efficiency of the sparse noise approach, where we could map RFs with both techniques we observed a good correlation in terms of RF position and diameter (Figure S4H). Thus, although the relationship between RF diameters observed under the two approaches was not perfect (Figure S4H), there was no systematic difference between the two approaches (mean difference: 1.1±0.6°; paired t-test; $P>0.05$).

To probe for more sluggish changes in cell firing evoked by stimuli in binocular visual space we also presented low frequency (0.2Hz) reversing gratings. These consisted of horizontally orientated black and white bars of various spatial frequencies (~0.2,0.1,0.08 and 0.04 cycles/°, + full screen flashes) and were delivered, as above, under both binocular and monocular viewing conditions.

To ensure truly monocular presentation of all stimuli above, where appropriate, irradiance at the non-stimulated eye was held constant (effective irradiance: 14 log photons/cm^2/s) using the same apparatus used to deliver full field stimuli. Background irradiance in the

experimental room was 13.6 log effective photons/cm^2/s (based on rod sensitivity; M-opsin = 13.7, S-opsin = 11.8, Melanopsin 13.5 log effective photons/cm^2/s).

Consistent with previous reports that eye-movements are minimal in anaesthetised mice [S10], these are extremely unlikely to have had a significant influence on the estimates of spatial response properties reported here. Specifically, we assessed this possibility by looking for coordinated changes (mean absolute difference) in receptive field position and size among simultaneously recorded cells. Firstly we compared these properties as determined by our responses to flashing bars under monocular and binocular viewing conditions (mapping sessions occurring 45-90min apart). To avoid confounding effects of binocular interactions, we restricted this analysis to monocular cells and found evidence of only very minimal changes in eye position (azimuth: 1.5±0.3; elevation: 2.0±0.5; diameter: 1.6±0.4; mean±SEM from 8 viewing conditions with 4 or more monocular cells). Secondly, we looked for coordinated changes in properties measured using bars vs. sparse noise (mapping sessions ~20min apart). Here we included all responsive cells regardless of functional class and found similarly minimal differences (azimuth: 2.1±0.5; elevation: 1.3±0.3; diameter: 1.7±0.4; mean±SEM from 9 viewing conditions with 4 or more cells). See also analysis in Figure S4C, indicating that we detect both large and small RFs among simultaneously recorded cells.

Histology
At the end of each experiment, mice were perfused transcardially with 0.1M phosphate buffered saline followed by 4% paraformaldehyde. The brain was removed and post-fixed in 4% paraformaldehyde for 30min and subsequently cryoprotected in 30% sucrose. The following day, brains were sectioned at 100µm on a freezing sledge microtome and either mounted directly onto slides (wildtype mice) using Vectasheild (Vectorlaboratories Ltd., Peterbourogh, UK) or first processed for X-gal staining (*Opn4*$^{+/tau\text{-}lacZ}$) as described below.

X-gal staining was performed as previously described [S1]. Brain sections were washed twice for 10 minutes each in buffer B (0.1M PBS at pH 7.4, 2mM $MgCl_2$, 0.01% Na-desoxycholate and 0.02% IGEPAL). Sections were then incubated for 18 hours in staining solution [buffer B with potassium ferricyanide (5mM), potassium ferrocyanide (5mM) and X-gal (Bioline Reagents Ltd, UK; 1mg/ml)] at 37°C in darkness. Following staining, sections were washed twice for 5 minutes in 0.1M PBS and mounted to slides using Vectashield as above.

After mounting DiI-labelled probe placements were visualised under a fluorescent microscope (Olympus BX51) with appropriate filter sets and, where appropriate, X-gal staining was visualised by standard light microscopy. Resulting images were then scaled to account for shrinkage (based on the known distance between electrode shanks) and aligned with appropriate stereotaxic atlas figures [S3] using the position and orientation of the optic tract and IGL (as revealed by X-gal staining) as landmarks. Anatomical locations of recorded cells were then estimated from these images, based on the known geometry of the electrode array. Consistent with previous modelling work [S14], we think it very unlikely that any isolated unit was located more than 50µm away from the recording site where we observe the largest spike amplitudes. Hence, we never observed single unit spikes that crossed our spike detection threshold on more than two channels of the 50µm spaced recording array. Similarly, we always observed a very sharp cut-off in visually evoked multiunit firing at electrode sites estimated (from our dye labelling) to lie outside the borders of the LGN (Figure S1A-B).

To produce maps of cell type densities, we first assigned cells to one of 3 anatomical templates corresponding to rostral, mid or caudal LGN. The small number of visually unresponsive cells we encountered were excluded this analysis. We then calculated the relative proportion of each visually responsive cell type using a moving circular window

(radius 100μm; step size 50μm) and smoothed the resulting maps by cubic spline interpolation. A similar procedure was used to produce maps of LGN retinotopic organisation except in this case, to increase sampling coverage, we mapped positions onto a single LGN template (correcting anatomical coordinates relative to the medial and dorsal borders of the LGN for each probe placement).

Supplemental References

S1. Hattar, S., Kumar, M., Park, A., Tong, P., Tung, J., Yau, K.W., and Berson, D.M. (2006). Central projections of melanopsin-expressing retinal ganglion cells in the mouse. J Comp Neurol *497*, 326-349.

S2. Brown, T.M., Gias, C., Hatori, M., Keding, S.R., Semo, M., Coffey, P.J., Gigg, J., Piggins, H.D., Panda, S., and Lucas, R.J. (2010). Melanopsin contributions to irradiance coding in the thalamo-cortical visual system. PLoS Biol *8*, e1000558.

S3. Paxinos, G.F., K.B.J. (2001). The mouse brain in stereotaxic coordinates: Second Edition, (San Diego: Academic Press).

S4. Govardovskii, V.I., Fyhrquist, N., Reuter, T., Kuzmin, D.G., and Donner, K. (2000). In search of the visual pigment template. Vis Neurosci *17*, 509-528.

S5. Jacobs, G.H., and Williams, G.A. (2007). Contributions of the mouse UV photopigment to the ERG and to vision. Doc Ophthalmol *115*, 137-144.

S6. Applebury, M.L., Antoch, M.P., Baxter, L.C., Chun, L.L., Falk, J.D., Farhangfar, F., Kage, K., Krzystolik, M.G., Lyass, L.A., and Robbins, J.T. (2000). The murine cone photoreceptor: a single cone type expresses both S and M opsins with retinal spatial patterning. Neuron *27*, 513-523.

S7. Sterratt, D.C., Lyngholm, D., Willshaw, D.J., and Thompson, I.D. (2013). Standard anatomical and visual space for the mouse retina: computational reconstruction and transformation of flattened retinae with the Retistruct package. PLoS Comput Biol *9*, e1002921.

S8. Zhao, X., Liu, M., and Cang, J. (2013). Sublinear binocular integration preserves orientation selectivity in mouse visual cortex. Nat Commun *4*, 2088.

S9. Montemurro, M.A., Senatore, R., and Panzeri, S. (2007). Tight data-robust bounds to mutual information combining shuffling and model selection techniques. Neural Comput *19*, 2913-2957.

S10. Grubb, M.S., and Thompson, I.D. (2003). Quantitative characterization of visual response properties in the mouse dorsal lateral geniculate nucleus. J Neurophysiol *90*, 3594-3607.

S11. Piscopo, D.M., El-Danaf, R.N., Huberman, A.D., and Niell, C.M. (2013). Diverse visual features encoded in mouse lateral geniculate nucleus. J Neurosci *33*, 4642-4656.

S12. Brainard, D.H. (1997). The Psychophysics Toolbox. Spat Vis *10*, 433-436.

S13. Pelli, D.G. (1997). The VideoToolbox software for visual psychophysics: transforming numbers into movies. Spat Vis *10*, 437-442.

S14. Pettersen, K.H., and Einevoll, G.T. (2008). Amplitude variability and extracellular low-pass filtering of neuronal spikes. Biophys J *94*, 784-802.
